# Integrating conventional and remote sensing with DC resistivity datasets to map groundwater potential areas using the analytical hierarchy process method, North Wadi Diit, Egypt

**DOI:** 10.1038/s41598-025-94598-7

**Published:** 2025-04-14

**Authors:** Mohamed Genedi, Noura Gouhar, Gad El-Qady, Ibrahim Gaafar, Ahmed El Mahmoudi

**Affiliations:** 1 Department of Geology, Faculty of Science, Mansoura University, Mansoura, 35516 Egypt; 2 National Research Institute of Astronomy and Geophysics (NRIAG), Helwan, 11722 Egypt; 3 Exploration Division, Nuclear Materials Authority, P.O. Box 530, Maadi, Cairo Egypt

**Keywords:** Conventional and remote sensing datasets, Analytical hierarchy process (AHP), Groundwater potential zones (GWPZ) mapping, DC resistivity data, Constrained and 2D inversions, Quaternary and fractured basement aquifers, Geophysics, Hydrogeology

## Abstract

This study investigates the groundwater potential (GWP) in Wadi Diit, an arid region with promising resource development prospects, by integrating topographic, hydrogeological, and mineralogical parameters. To analyze the study area, a combination of conventional methods, remote sensing data from Sentinel-2, ASTER-GDEM, and ASTER-L1B, as well as DC resistivity datasets was utilized. The study region comprises Precambrian, Tertiary, and Quaternary surface rock units, supporting lithosol and Yermosol soil types. Barren lands dominate the landscape, while the southern portion experiences higher rainfall. Nine thematic layers (quartz index, carbonate index, slope, rainfall, drainage density, topographic wetness index, lineament density, land cover, and mafic index) were classified and weighted using GIS-based analytical hierarchy process, achieving a model accuracy of 0.0959. The GWP zones were categorized into very low (4.53%), low (17.33%), moderate (27.05%), high (27.79%), and very high (23.3%) categories, predominantly falling within moderate to very high classifications. Validation through hydrogeological data from 11 wells and a receiver operating characteristic curve analysis (area under curve = 0.8) confirmed the model’s reliability. DC resistivity measurements were conducted at nine vertical electrical sounding (VES) sites using a Schlumberger array (AB/2 = 500 m) along two profiles. The data were analyzed using various inversion techniques, including unconstrained 1D-VES, laterally constrained inversion (LCI-VES), spatially constrained inversion (SCI-VES), and 2D-VES inversions. A 0.3 constraint factor was applied to assess the accuracy of the model parameters, as their STDF derived from SCI-VES data were determined to be well-resolved. The SCI-VES and 2D-VES inversion results identified four distinct geological layers; unconsolidated surface deposits, gravelly-sand sediments of fresh-brackish Quaternary aquifer (30–384 Ω m and 3.7–15.9 m depth), saturated clayey-sand deposits, and saline Fractured Basement aquifer (10–137 Ω m and 33–90.4 m depth). The region exhibits a complex geological structure, characterized by an uplifted Fractured Basement aquifer trending southeast and southwest as indicated by 2D-VES models. The north-central region emerges as the most favorable location for substantial GWPZ, making it strategically ideal for the installation of additional water wells.

## Introduction

The over-exploitation of groundwater and climate change have significantly strained global groundwater resources, necessitating comprehensive assessments of aquifer productivity and water potential. In Egypt, one of the world’s most water-scarce countries, this challenge is compounded by a 40-billion-cubic-meter annual water deficit^1^, which is projected to worsen due to factors such as the Grand Ethiopian Renaissance Dam, urban expansion in arid coastal regions, and climatic shifts. With only 5% of its land habitable, primarily along the Nile Valley and Delta, the country relies heavily on the Nile for freshwater. However, rapid population growth (tripling over five decades) has intensified water demand while agricultural land loss exacerbates food insecurity. To bridge the widening gap between water availability and food supply, strategies must focus on expanding agriculture beyond traditional areas into currently restricted zones, balancing resource use with sustainability^2^.

Sustainable development in Egypt’s deserts hinges on horizontal expansion and the strategic utilization of non-conventional water resources, particularly groundwater. The Eastern Desert, notably the Southeastern region, offers significant potential for land reclamation, mineral extraction^3^, and tourism due to its rich ore deposits and unique geological features. However, the scarcity of fresh water in coastal cities like Shalateen constrains resource exploitation, necessitating reliance on groundwater as a primary water source. While desalination is an alternative in remote areas such as Halayeb, its high cost reinforces the importance of groundwater from complex aquifers, which may have been replenished by past rainfall and flooding events. Effective water security and resource management require the identification of groundwater potential zones (GWPZs) and the implementation of watershed planning to harness stored water. The target site’s favorable geological and topographical characteristics, including shallow Quaternary and fractured basement aquifers, enhance its capacity for groundwater availability, underscoring the interdependence of water resource management and sustainable development in these regions.

Groundwater availability in semi-arid regions exhibits significant spatiotemporal variability, influenced by geological features, basin slope, low precipitation, high temperatures, evaporation rates, and land-use/land-cover (LULC) changes^4,5^. These interdependent factors complicate the assessment of water resources, underscoring the importance of delineating groundwater potential zones (GWPZs). Identifying GWPZs enables more precise investigations into water resources, facilitating a comprehensive understanding of their sustainable long-term management.

**Fig. 1:**
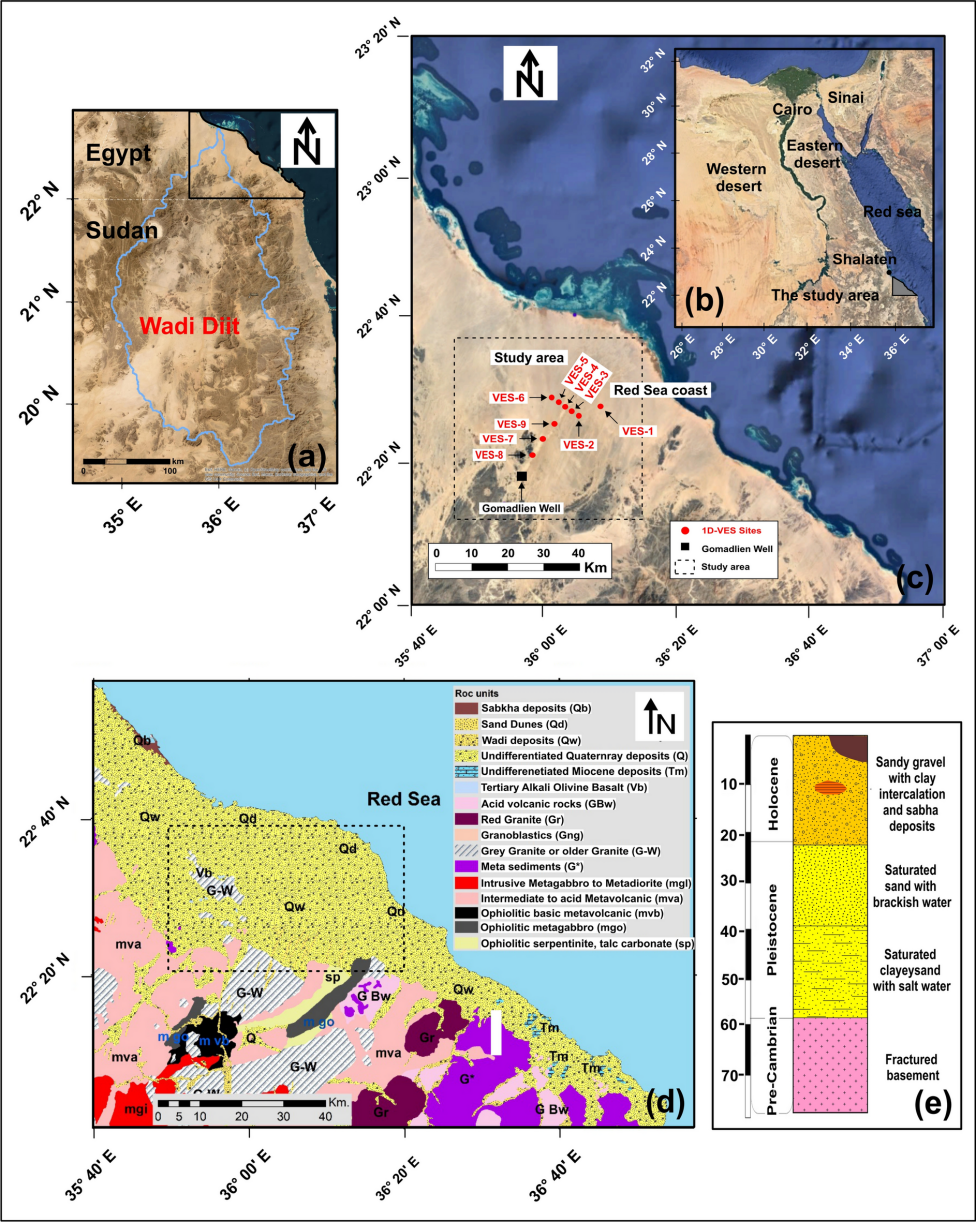
location map of Wadi Diit basin (**a**), Google earth map of Egypt (**b**), location map of the study area including location of VES-DC sites, and nearest neighbor well (**c**), geological map of the study area (**d**), and lithe-staratigraphic column of the study area (**e**). (The satellite images of the study area were obtained from Google Earth Pro 7.3 (https://www.google.com/earth/about/versions/) while the figure was generated using Surfer 13.6 (www.goldensoftware.com) by Mohamed Genedi, Noura Gouhar and Gad El-Qady).

The coastal plain between Shalateen and Abu Ramad, particularly Wadi Diit in Egypt’s Eastern Desert, presents significant potential for development and reclamation due to its strategic location, potentially attracting government investment for urban expansion and tourism (Fig. 1a). Extending approximately 85 km within Egypt (Fig. 1b) and up to 300 km into Sudan, Wadi Diit occupies a critical geographical position. The study area, situated between latitudes 22° 27′–22° 41′ N and longitudes 36° 1′–36° 18′ E (Fig. 1c), exhibits diverse topography, with rugged terrain in the west transitioning to gentle slopes toward the Red Sea coast in the east. This semi-arid to arid region is projected to experience increased frequency of prolonged droughts, intermittent rainfall, and severe flooding. Recent studies^6–8^ have explored groundwater presence in Wadi Diit’s delta, underscoring its importance for sustainable development amidst climatic challenges*.*

Remote sensing and GIS technologies have revolutionized water resource modeling, particularly in arid regions, by enabling the efficient processing and analysis of spatial data. These tools facilitate the rapid identification of potential groundwater zones across extensive areas, offering cost-effective solutions for hard-to-reach locations through high-resolution satellite imagery. The integration of remote sensing with GIS provides a robust framework for hydrogeological mapping and groundwater accumulation assessment. In this study, Esri Sentinel-2, ASTER-DEM, and ASTER-L1B datasets were utilized to analyze land cover, topographic characteristics, hydrogeological features, and mineral compositions, including quartzite, carbonate, mafic, and ultramafic rocks, thereby demonstrating the interdependence of these methodologies in advancing hydrogeological understanding.

The Analytic Hierarchy Process (AHP) serves as a robust tool for addressing complex multi-criteria decision-making (MCDS) problems in groundwater studies by facilitating pairwise comparisons of spatial parameters, weighted according to expert judgment^9^. This approach has been effectively integrated with remote sensing and GIS techniques by various researchers^5,10–14^ to systematically map groundwater potential areas (GWRP).

Groundwater presence, movement, and quality are significantly influenced by geological structures such as faults and dykes, as well as processes like saltwater intrusion. This proposal employs the continuous electrical DC resistivity method to investigate shallow subsurface layers, assess water quality, detect saltwater intrusion, and determine basement rock depths. To analyze the collected data, both unconstrained and constrained inversion techniques were utilized. In semi-stratified sedimentary regions, one-dimensional unconstrained inversions often produce inconsistent results due to noisy data and model equivalence^15^, although these are only marginally affected by 2D and 3D fault-related effects^16^. To enhance lateral resolution and address these limitations, constrained inversion methods, including Laterally Constrained Inversion (LCI) and Spatially Constrained Inversion (SCI), were applied, providing more reliable interpretations of the subsurface structure.

In conventional one-dimensional inversion, constraints are imposed on the output parameters (resistivity, thickness, and depth) of adjacent models during the inversion process. LCI, a profile-oriented method, imposes constraints along a scan line to ensure parameter continuity between adjacent one-dimensional models without interaction between lines^17^, favoring structures aligned with the profile direction. SCI employs the least squares method, adapted for inversion in terrains that are nearly three-dimensional and layered, with structures defined by spatial constraints. This approach ensures smooth lateral transitions between layers^18,19^. The primary distinction between these techniques lies in their constraint application: LCI restricts adjustments laterally along the profile, while SCI operates in two dimensions along and across the profiles^19^, enhancing its ability to capture complex subsurface geometries. LCI selectively prioritizes structures aligned with the profile direction, whereas SCI emphasizes features both parallel and perpendicular to these profiles^17–19^. To validate the reliability of inverted models, sensitivity analyses are conducted on the final parameters derived from constrained inversions^20^. These methods have been widely applied in electrical and electromagnetic studies, as evidenced by numerous researchers^16,17,19,21–27^ who have demonstrated their efficacy in improving model accuracy and resolving subsurface structures.

This study integrates remote sensing and geophysical techniques to evaluate GWPZ for sustainable water resource management in the region, addressing demands for drinking water, industry, and agriculture. A weighted overlay analysis using the AHP method was employed to combine nine thematic layers (quartz index, carbonate index, slope, rainfall, drainage density, topographic wetness index, line density, land use/land cover, and mafic index) into a comprehensive GWPZ map. Additionally, the research investigates the efficacy of constrained inversion methodologies, specifically LCI and SCI, to enhance the resolution of subsurface boundaries between geological units. These techniques address the non-uniqueness issues inherent in one-dimensional unconstrained inversion results, thereby improving model accuracy and facilitating more reliable subsurface characterization. The interdependence of these approaches underscores their collective potential in advancing groundwater exploration and management strategies.

## Geological and hydro-geological setting

Figure 1e presents a generalized stratigraphic column for Halaib and Shalateen^28^ while Fig. 1d illustrates the geological units across the study area^29^. The western portion of the study area is dominated by Precambrian rocks, which include ophiolitic assemblages, island arc assemblages, and early to post-magmatic units. Ophiolitic rocks consist of intensely tectonized serpentinites, talc-carbonate formations, and metagabbros, while the island arc assemblage comprises meta-volcano-sedimentary rocks, meta-volcanics, and gabbro-diorites. Magmatic activity is evidenced by the integration of older and younger granites^30^. The Phanerozoic sedimentary rocks in the studied region comprise Tertiary basalt, Miocene sediments, and Quaternary sediments^30^. Miocene and Quaternary sediments unconformably overlie the bedrock, while Tertiary basalt forms isolated hills of low relief on the Piedmont plain at the base of the Red Sea mountain shield. These basaltic formations, primarily linked to Tertiary volcanic activity in the Red Sea shelf, occasionally intersect biotite granite and are associated with Graben structures^30^. Isolated Miocene hills, located west of the Abu Ramad-Hala’ib region, reveal the presence of Miocene sediments, which are predominantly composed of alternating limestone and marl layers interbedded with claystone. These sequences are notably ferruginous, reflecting specific depositional conditions^30^. The hills, obscured by gravel deposits and rock fragments sourced from the surrounding terrain. The study area features prominent Miocene sedimentary rock outcrops, while alluvial deposits characterize the Pleistocene and recent ages. Quaternary deposits are evident in the valley floor’s alluvial fills, sand dunes, sand sheets, alluvial fans, and sabkhas extending from west to east on the Red Sea coastal plain. These deposits stretch along the Red Sea coast, spanning from south of Shalateen city in the north to Abu Ramad city in the south^30^.

The studied area, situated along the Red Sea coast, exhibits a complex tectonic framework shaped by multiple fault systems and dykes formed during various episodes of Red Sea rifting. Notably, both shallow and deep-rooted faults traverse the subsurface of the Shalateen region to the north^28^. The predominant fault systems include meridional (N-S), Mediterranean (E-W), Gulf of Suez, Red Sea (NW–SE), Aqaba (NNE-SSW), Northeast Africa-Southwest, and Syrian Arc (ENE) trends, collectively reflecting the region’s intricate structural interdependencies.

The geomorphology of the Shalateen-Halaib area in the Eastern Desert is characterized by three primary units: the Red Sea coastal plain, the watershed lands, and the watershed area. The coastal plain extends parallel to the Red Sea coast in a northwest-southeast orientation, while the watershed lands comprise mountainous regions with drainage basins and sandy plains. The watershed area, dominated by igneous and metamorphic rocks, broadens southward^31^. Transitioning west to east, the region exhibits high mountains associated with the Arabian-Nubian Shield, isolated hills composed of metavolcanic, metasedimentary, sedimentary, and Tertiary volcanic rocks, a plateau plain, alluvial fans, and finally, the coastal plains. These features reflect intricate interdependencies among geological composition, topography, and hydrological processes.

The morphometric parameters of drainage basins in the Eastern Desert significantly influence their hydrological characteristics, enabling classification into three distinct categories^31^. Class I wadis, characterized by low flow rates, low drainage density, and high branching ratios, exhibit strong potential for recharging shallow aquifers, which could serve as vital water resources. In contrast, Class II wadis, marked by low branching ratios, high flow frequency, and high drainage density, demonstrate the highest flood risk but minimal groundwater potential. Class III wadis, with moderate values for drainage density, flow frequency, and branching ratio, balance both groundwater recharge and flood potential, highlighting the interdependence of these hydrological features^31^.

The Shalateen region hosts two primary aquifers: the Quaternary aquifer and the fractured basement aquifer. The Quaternary aquifer, composed of sand, gravel, silt, and clay, is located in coastal plains, piedmont areas, and valley channels, with a maximum thickness of 15 m, overlain by Nubian sandstone or limestone. Its groundwater lies at shallow depths, often influenced by saltwater intrusion^32^, particularly in salt marshes at the surface. Seasonal variations in salinity, peaking at summer’s end, are evident in both aquifers. The basement aquifer’s hydraulic properties, including transmissivity and water depth (ranging from 1 to 27 m)^32^, depend on fracture distribution and geological complexity. Salinity levels vary spatially, with higher values near metasediment and metavolcanic deposits, while granitic and ophiolite rocks correlate with lower salinity. Recharge occurs primarily through sporadic rainfall and surface runoff during the rainy season, infiltrating via soil or fractures to replenish the water table, with greater rates observed in piedmont and coastal plains compared to major valleys^33^.

**Fig. 2:**
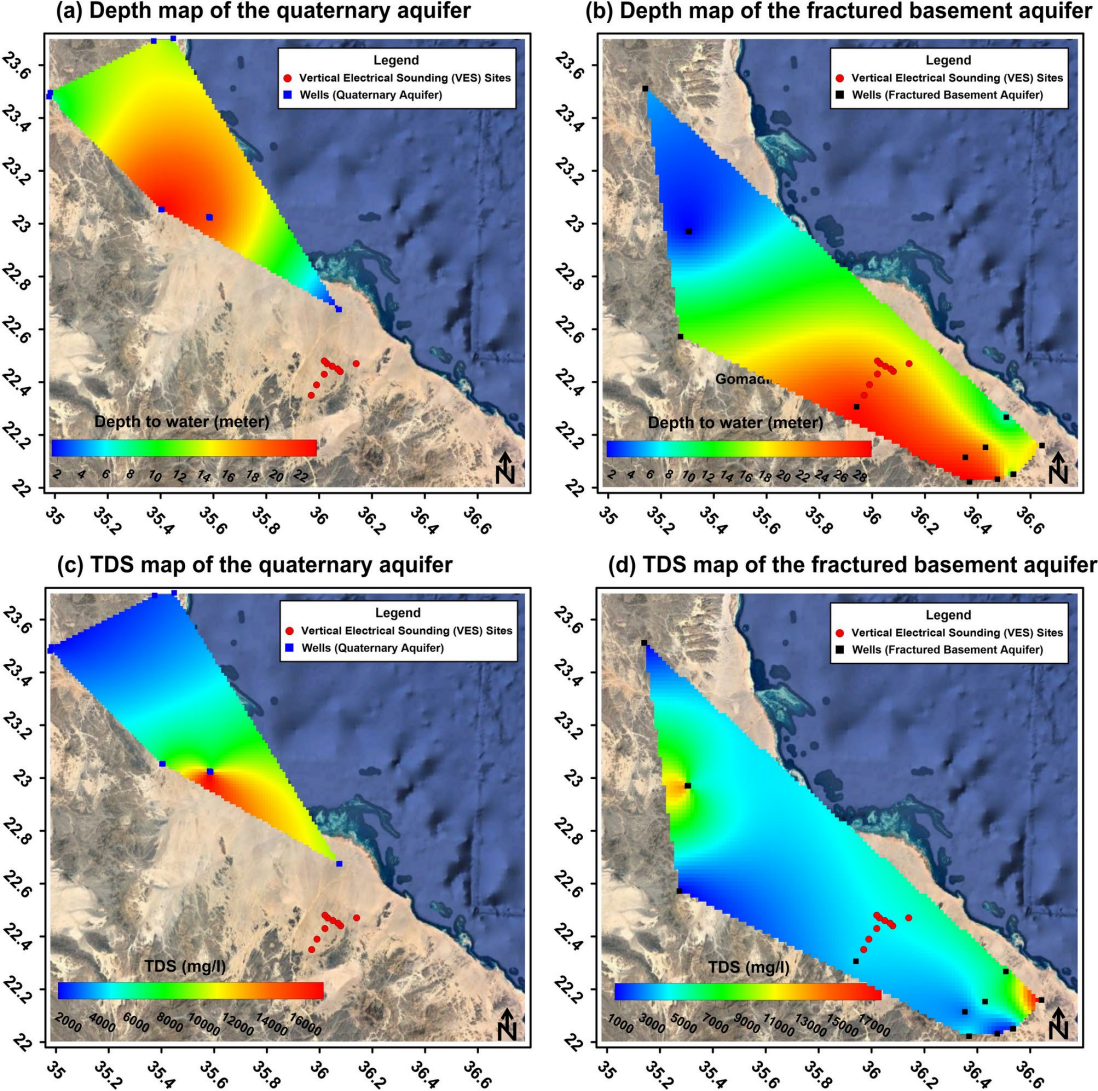
Depth to top map of the Quaternary groundwater aquifer (**a**), depth to top map of the fractured basement aquifer (**b**), TDS map of the quaternary aquifer (**c**), and TDS map of the fractured basement aquifer (**d**). (The satellite images of the study area were obtained from Google Earth Pro 7.3 (https://www.google.com/earth/about/versions/) while the figure was generated using Surfer 13.6 (www.goldensoftware.com) by Mohamed Genedi and Noura Gouhar).

Groundwater samples were collected from 25 sites across two major aquifers within the study area and its surroundings^34^. The Quaternary aquifer, accessed via 11 wells (Fig. 2a) with water depths ranging from 1.05 to 23.4 m, exhibited salinity levels classified as saline to brackish (Fig. 2c), varying between 1253 mg/L (well No. 4) and 18,854 mg/L (well No. 8). In contrast, the fractured basement aquifer, explored through 14 hand-dug wells (Fig. 2b) with depths of 1.4 to 29.5 m, demonstrated a broader range of salinity (Fig. 2d), with total dissolved solids ranging from 320 mg/L (well No. 24) to 19,375 mg/L (well No. 28). While most samples from this aquifer were categorized as fresh to brackish, two wells (No. 28 and No. 37) were identified as saline^34^.

## Data and methods

**Fig. 3:**
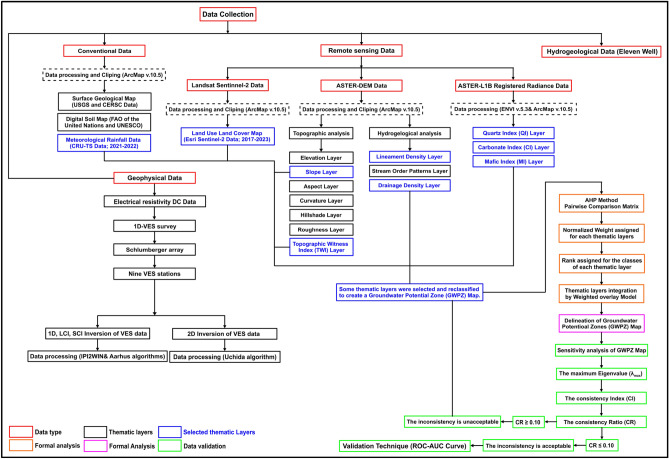
The flowchart of the methodology of conventional, remote sensing and DC resistivity datasets adopted in this study. (The flowchart was drawn using Surfer 13.6 (www.goldensoftware.com) by Mohamed Genedi and Gad El-Qady).

To identify potential groundwater sustainability zones in the study area, both conventional digital datasets and satellite remote sensing data were utilized. Figure 3 presents a flowchart illustrating the data and methodologies employed to detect groundwater potential zones (GWPZ). Remote sensing data analysis was conducted using ENVI (Version: 5.3, URL link: https://www.nv5geospatialsoftware.com/) and ArcMap (Version: 10.5, URL link: https://www.esri.com/) software. Selected scenes were extracted to cover the study area exclusively, then displayed and plotted in the WGS 1984/UTM Zone (36 North) coordinate system with a resampling resolution of 15 × 15 m, ensuring spatial accuracy and consistency.

### Conventional datasets

Geology significantly influences groundwater potential through the hydraulic properties of rocks, which govern water infiltration and seepage^35^. Concurrently, soil types regulate infiltration rates into subsurface formations, directly impacting groundwater recharge processes^10,36^. In arid and semi-arid regions, rainfall constitutes the primary source of groundwater recharge, with higher precipitation levels generally correlating to greater water availability.

The analysis incorporated conventional datasets comprising surface geological, digital soil, and annual rainfall maps, highlighting their interdependent roles in providing a comprehensive foundation for assessment. A geological map of the study area, derived from surface geological maps of Africa provided by the United States Geological Survey (USGS) and the Central Energy Resources Data Management Services (CERSC) project^37^, was utilized to delineate distinct geological units (https://www.sciencebase.gov/catalog/item/60d0ff26d34e86b938aab404). Digital soil data, sourced from the Food and Agriculture Organization (FAO) of the United Nations and the UNESCO Soil Map of the World^38^, facilitated the creation of a 1:5,000,000 scale world soil map (https://data.apps.fao.org/map/catalog/srv/eng/catalog.search#/metadata/446ed430-8383-11db-b9b2-000d939bc5d8). Mean annual precipitation estimates for 2021–2022 were obtained using high-resolution gridded datasets (0.5 degree resolution) from the Climatic Research Unit (CRU), using the website link (https://crudata.uea.ac.uk/cru/data/hrg/cru_ts_4.08/cruts.2406270035.v4.08/pre/).This research was supported by the UK Natural Environment Research Council (NERC), the US Department of Energy, and the UK National Centre for Atmospheric Science (NCAS).

## Remote sensing datasets

### Land use land cover (LULC) data

Land use and land cover (LULC) analysis evaluates spatial distribution and terrain characteristics, offering critical insights into hydrological processes such as water infiltration, soil moisture, and surfacewater and groundwater^39^. Seasonal variations in LULC significantly influence groundwater storage and recharge dynamics^40^. For this study, high-resolution (10-m) annual LULC datasets spanning 2017–2023 were sourced from Esri Sentinel-2 Land Use/Land Cover maps (https://livingatlas.arcgis.com/landcoverexplorer).

### ASTER-GDEM data

The Advanced Spaceborne Thermal Emissions and Reflection Radiometer (ASTER) datasets, specifically the global digital elevation model (GDEM) and Level 1B (L1B) data, were utilized for this study. These ready-made datasets were sourced from NASA’s Earth Data Search website (https://search.earthdata.nasa.gov/search). The ASTER-30 m DEM data facilitated the extraction of topographic and morphometric characteristics of the study area. Mineralogical indicators, including quartz, carbonate, and mafic components, were derived from the ASTER-L1B data, highlighting their interdependent roles in geological analysis.

### Topographical analysis of ASTER-GDEM data

Topographic analyses utilizing ASTER-DEM data incorporate parameters such as elevation, slope, slope aspect, curvature, hillshade, roughness index, and topographic wetness index, all of which are crucial for environmental management, particularly in GWPZ assessments.

Elevation and slope serve as primary indicators, with plain areas typically exhibiting higher infiltration rates compared to moderate and high-elevation regions^41^ due to their geomorphological characteristics. Slope characteristics significantly influence surface water infiltration, groundwater recharge, and hydrological processes. Flat or gentle slopes enhance infiltration and groundwater recharge due to reduced runoff, whereas moderate to steep slopes increase surface runoff and diminish recharge potential^14^. Weighting schemes assign higher values to gentle slopes with thicker soil layers and lower values to steep slopes^42^.

Slope aspect, defined as the horizontal direction of maximum gradient^43^ measured clockwise from north^44^, affects sunlight exposure and moisture availability. South-facing slopes in the northern hemisphere receive more direct sunlight, leading to drier conditions, while north-facing slopes retain more moisture and water resources. This variability is particularly pronounced in regions north of the equator, where north- and east-facing slopes exhibit greater water availability compared to south- and west-facing slopes.

The curvature of the Earth’s surface, which quantitatively describes its profile, is categorized into flat, concave-up, and convex-up regions^45^ and significantly influences water accumulation and infiltration capacity^46^. Concave and flat areas facilitate greater water retention and infiltration from rainfall compared to convex regions. High curvature values are assigned greater weight in analyses, reflecting their enhanced capacity for water resource capture, while lower values correspond to reduced potential^46^*.* Hillshading, a grayscale 3D surface representation incorporating solar position^47^, calculates illumination values (0–255) based on sun azimuth and altitude, enhancing terrain visualization. Additionally, the roughness index (RI), which quantifies elevation differences between adjacent cells in a digital elevation model (DEM), reflects terrain undulation that characterizes a mountainous area^45^, with higher values indicating greater irregularity. The Topography Wetness Index (TWI) quantifies the influence of terrain on hydrological processes, particularly groundwater infiltration, by integrating upslope contributing area (α) and topographic gradient (β) through the formula (TWI = ln α/tan β). TWI values are notably higher in plain areas prone to frequent flooding within the basin^48^, directly correlating with increased groundwater potential.

### Hydrogeological analysis of ASTER-GDEM data

Hydrogeological analysis incorporates key parameters such as stream networks, lineament density, and drainage density patterns to assess groundwater potential. Stream networks are derived from Digital Elevation Models (DEMs) by applying an area threshold to the flow accumulation raster, identifying cells with flow values exceeding this threshold. Lineament and drainage densities were calculated using the Line Density tool within the Spatial Analyst extension of ArcMap.

Lineaments, extracted from hillshade maps, represent structurally controlled features like fractures and faults^35^, which enhance secondary porosity and permeability^39,42^. These linear elements significantly influence groundwater recharge and occurrence, with higher potential typically observed in proximity to lineaments. Conversely, groundwater potential decreases with increasing distance from these structural features^10^, underscoring their critical role in hydrogeological assessments. Lineament density serves as a key indicator of potential groundwater recharge, suggesting a higher likelihood of replenishment in areas with significant linear features^14^. Groundwater potential is closely linked to lineament density, with high-density areas indicating greater potential and thus receiving higher weighting, while low-density areas suggest lower potential and are assigned lower weights^10,42,49^.

Drainage density, which reflects the extent of stream channel networks within a basin, plays a crucial role in groundwater availability and contamination^50^, influenced by factors such as lithology, slope angle, vegetation, and soil type that collectively affect infiltration rates. Notably, a negative correlation exists between drainage density and both slope characteristics and the hydraulic properties of geological units^51^, underscoring their interdependent influence on groundwater systems. Drainage density, defined as the total length of streams in a drainage basin divided by its area, serves as a critical parameter for assessing groundwater potential^39,52^. It exhibits an inverse relationship with soil permeability and infiltration capacity; high drainage density signifies impermeable soils on steep slopes, leading to reduced infiltration and diminished groundwater recharge. Conversely, low drainage density indicates greater permeability and infiltration, enhancing groundwater potential^10,42^. This inverse proportionality between drainage density and groundwater potential^14^ is pivotal in partitioning areas for groundwater assessment, *wherein lower densities are* assigned higher weights^42^ due to their favorable contribution to aquifer replenishment.

### ASTER-L1B data

Mineral indices, including quartz (QI), carbonate (CI), and mafic (MI), provide a systematic framework for lithological mapping in arid and semi-arid environments^53^. QI and CI maps identify quartz- and carbonate-rich deposits, which typically exhibit high permeability and significant groundwater potential. Conversely, the MI map highlights mafic and ultramafic rocks, characterized by low permeability and limited groundwater potential. These indices effectively discern the mineralogical composition of quartz, carbonate, and basic rock types^54^ through specific calculations*:* QI = (Band 11*Band 11)/(Band 10*Band 12), CI = (Band 13/Band 14), and MI = (Band 12 *Band 14^3^)/(Band 13^4^). The interdependence of these indices underscores their utility in differentiating geological features based on permeability and groundwater prospects.

### Weight calculation using analytical hierrarchy process (AHP) method

Geospatial techniques, combined with knowledge-based factor analysis, were employed to identify groundwater potential areas by evaluating multiple parameters. The analytical hierarchy process (AHP), a prominent multi-criteria decision-making (MCDM) method, was utilized to integrate diverse objective layers and determine their weighted significance^10,55^. Widely recognized for its effectiveness in spatial decision-making, AHP has been instrumental in addressing complex groundwater-related challenges^9,56–58^, demonstrating its robust applicability in this domain.

This method systematically decomposes the decision-making process into four interrelated steps. Initially, a pairwise comparison matrix (PCM; m*m) is constructed to prioritize criteria, where (m) represents the number of factors influencing the determination of GWR^59^. Using Saaty’s scale (1–9) as shown in Table 1, each element in the PCM is scored; a score of 1 is assigned when an item is compared to itself, while scores greater than 1 reflect comparisons with other items^59^. Subsequently, a normalized pairwise comparison matrix (NPCM) is generated by dividing each PCM value by the sum of its respective column^60,61^ (see Table 2). In the third step, normalized weights are calculated to minimize subjectivity^61^. This involves summing the rows of the NPCM to determine total weights for each variable and then normalizing these weights by dividing each row sum by the aggregate of all row sums, ensuring the total normalized weights equal one^60^. Finally, to assess the accuracy of the PCM matrix, the consistency index (CI), random index (RI), and consistency ratio (CR) were computed, taking into account the number of parameters.

**Table 1:** Saaty’s scale for Analytical Hierarchy Process (AHP) method^57,60^.

Intensity of Importance	Definition	Explanation
1	Equal importance	Two activities contribute equally to the objective
2	Weak or slight importance	When compromise is needed
3	Moderate importance	Experience and judgment slightly favor one activity over another
4	Moderate plus	When compromise is needed
5	Strong importance	Experience and judgment strongly favor one activity over another
6	Strong plus	When compromise is needed
7	Very strong importance	An activity is favored very strongly over another; its dominance is demonstrated in practice
8	Very, very strong	When compromise is needed
9	Extreme importance	The evidence favoring one activity over another is of the highest possible order of affirmation
Reciprocals of above	If activity i has one of the above non-zero numbers assigned to it when compared with activity j, then j has the reciprocal value when compared with i

**Table 2:** Pairwise Comparison Matrix (PCM) of nine variables for AHP method.

Variable	QI	CI	Slope	Rainfall	DD	TWI	LD	LULC	MI
QI	1	2	2	3	5	7	8	9	9
CI	0.5	1	2	2	4	6	7	8	8
Slope	0.5	0.5	1	2	4	6	7	8	8
Rainfall	0.33	0.5	0.5	1	3	5	6	7	7
DD	0.2	0.25	0.25	0.33	1	3	3	4	5
TWI	0.14	0.17	0.17	0.2	0.33	1	3	4	4
LD	0.13	0.14	0.14	0.17	0.33	0.33	1	3	3
LULC	0.11	0.13	0.13	0.14	0.25	0.25	0.33	1	2
MI	0.11	0.13	0.13	0.14	0.2	0.25	0.33	0.5	1
Total	3.02	4.81	6.31	8.99	18.12	28.83	35.67	44.5	47

The Eigen vector (Vp) for each row is determined using the formula (Vp = W_1_*W_2_*…..*W_n_)^(1/n)), where (n) represents the number of parameters in the analysis. The weighting coefficient (Cp) is subsequently derived by dividing each (Vp) value by the sum of all (Vp) values in its respective column. A consistency vector matrix is then obtained by multiplying the pairwise comparison matrix (PCM) with the weighting coefficient matrix (Cp). The eigenvalue (E) for each variable is calculated by dividing the corresponding consistency vector element by the associated weighting coefficient (Cp). Finally, the highest eigenvalue (λ_max_) of the matrix is computed as the average of all individual eigenvalues, expressed as (λ_max_ = E/n = (λ_1_ + λ_2_ + …..λ_n_)/n)^9^. To calculate λ^60^, each column of the PCM was first multiplied by its corresponding variable weight. The resulting values were then summed row-wise to derive the weighted sum. Finally, dividing this weighted sum by the variable weight yielded the value of λ^60^, thereby integrating the interdependent parameters into a cohesive computation process (Table 3).

**Table 3:** Normalized Pairwise Comparison Matrix (NPCM) of nine variables for AHP method.

Variable	QI	CI	Slope	Rainfall	DD	TWI	LD	LULC	MI	Total Weight	Norm. Weight
QI	0.33	0.42	0.32	0.33	0.28	0.24	0.22	0.2	0.19	2.53	0.28
CI	0.17	0.21	0.32	0.22	0.22	0.21	0.2	0.18	0.17	1.89	0.21
Slope	0.17	0.1	0.16	0.22	0.22	0.21	0.2	0.18	0.17	1.63	0.18
Rainfall	0.11	0.1	0	0.11	0.17	0.17	0.17	0.16	0.15	1.22	0.14
DD	0.07	0.05	0.04	0.04	0.06	0.1	0.08	0.09	0.11	0.63	0.07
TWI	0.05	0.03	0.03	0.02	0.02	0.03	0.08	0.09	0.09	0.44	0.05
LD	0.04	0.03	0.02	0.02	0.02	0.01	0.03	0.07	0.06	0.3	0.03
LULC	0.04	0.03	0.02	0.02	0.01	0.01	0.01	0.02	0.04	0.2	0.02
MI	0.04	0.03	0.02	0.02	0.01	0.01	0.01	0.01	0.02	0.16	0.02
Total	–	–	–	–	–	–	–	–	–	9	1

The consistency index (CI) was computed using the formula (CI = λ_max_^(1/n)/n). Subsequently, the consistency ratio (CR) was derived by dividing the CI by the random consistency index (RI), with RI values obtained from Saaty’s standardized Table 4^59,62^. A CR value of zero signifies perfect consistency in pairwise comparisons, while a value below 0.10 indicates acceptable consistency, allowing the analysis to proceed with a reasonably reliable judgment matrix. Conversely, a CR value exceeding 0.10 necessitates a review of the judgments to identify inconsistencies and implement necessary corrections, ensuring the integrity and validity of the analytical process^63,64^.

**Table 4:** Saaty’s ratio index of Random Consistency for different N values^57^.

n	1	2	3	4	5	6	7	8	9
RI	0	0	0.58	0.9	1.12	1.24	1.32	1.41	1.49

### Mapping of groundwater potential zones (GWPZes)

The weights assigned to each class in the thematic maps were determined based on their characteristics and water capacity potential using the Analytical Hierarchy Process (AHP) method. Feature classes were ranked and normalized via geometric mean criteria^65^, while sub-classes within each thematic class were rated on a scale of 1–5 according to their influence on groundwater potential^66^. These ratings corresponded to raster pixel values categorized as very low (1), low (2), medium (3), high (4), and very high (5).

The Groundwater Potential Zone (GWPZ) pixel values were subsequently calculated using the formula (GWPZ = ∑(W_A_ × R_B_)) where W_A_ denotes the weighted value of pixels in the variable thematic layers, and R_B_ represents the rank value of pixels in the subclasses of these layers^10,14^. Here, (A) signifies the total number of integrated thematic layers, and (B) indicates the number of subclasses within each ranked layer, highlighting the interdependence of these parameters in assessing groundwater potential.

### DC resistivity data acquisition, processing and inversion

A DC electrical resistivity survey was conducted at nine 1D Vertical Electrical Sounding (VES) sites along two profiles in the southwestern part of the study area, near faults downstream of the valley and along the Red Sea coast (Fig. 1c). The Schlumberger configuration, with a maximum electrode spacing (AB/2) of 500 m, was employed using an IRIS SYSCAL-R2 resistivity/IP meter for data acquisition. The locations of all VES sites are depicted in Fig. 1c. To enhance interpretation accuracy and minimize uncertainty, the collected apparent resistivity data were calibrated using prior hydrogeological datasets, including aquifer depth information from nearby wells. This integration of field measurements and existing data underscores the interdependence of geological context, survey design, and interpretative methods in understanding subsurface structures.

**Fig. 4:**
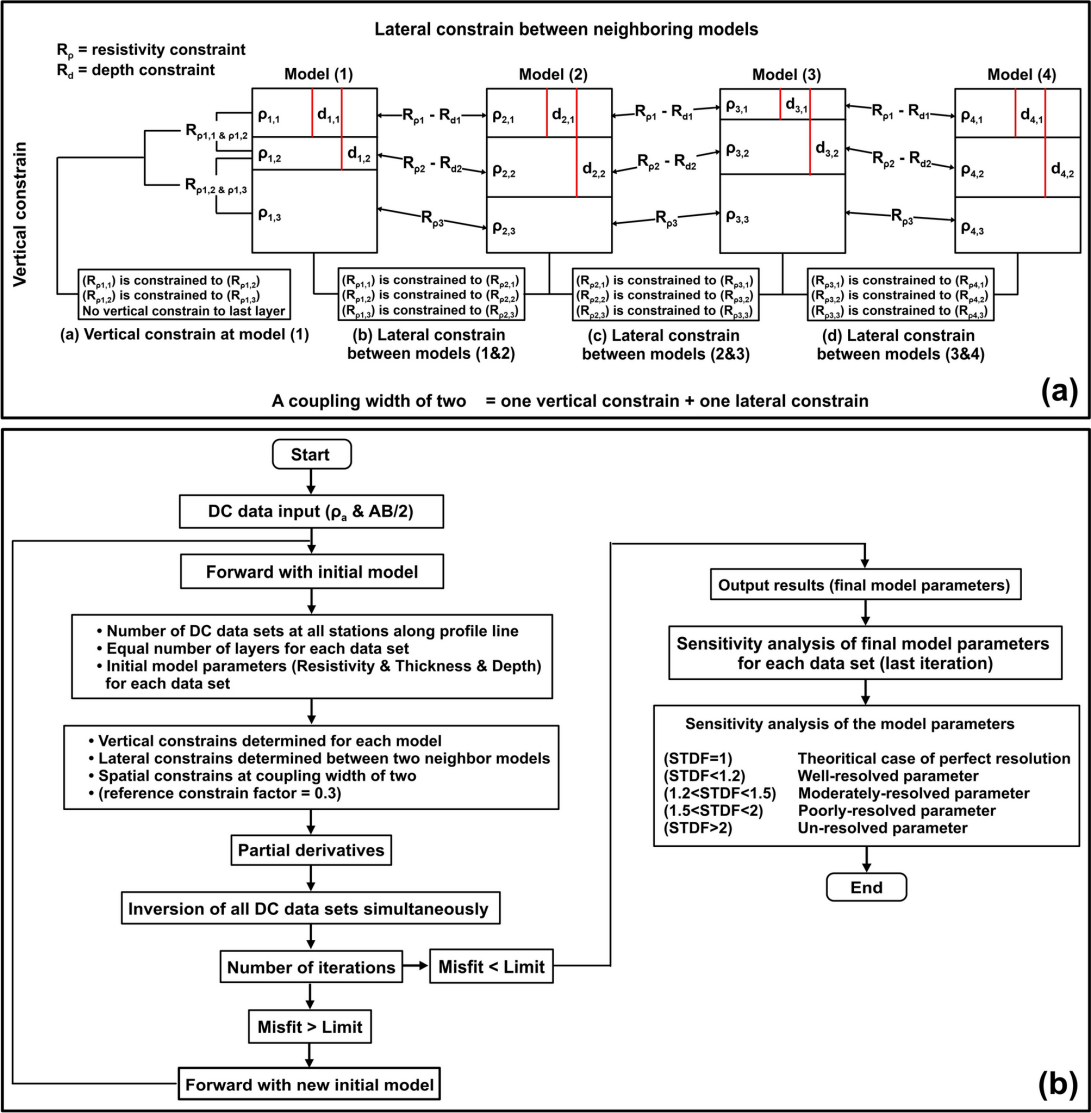
The flowchart of the methodology of the lateral and spatial constrain (**a**), and two dimensional inversion using fortran code (**b**) adopted to DC resistivity data in this study. (These flowcharts were drawn using Surfer 13.6 (www.goldensoftware.com) by Mohamed Genedi).

Constrained inversion techniques, including Laterally Constrained Inversion (LCI) and Spatially Constrained Inversion (SCI), were applied to the Vertical Electrical Sounding (VES) data using an inversion code developed by the Hydro-geophysics Group at Aarhus University, Denmark^67^ (AarhusInv algorithm, Version:8.11, URL link: https://hgg.au.dk/). These methods enhance the lateral resolution and smoothness of one-dimensional model parameters by imposing constraints on resistivity and thickness during the inversion process (see Fig. 4a, the flowchart of AarhusInv algorithm). Specifically, LCI and SCI inversions along two profiles utilized a constraint factor of 0.3 between adjacent VES sites, ensuring improved continuity and coherence in the resulting models. This approach effectively bridges the limitations of conventional one-dimensional inversions by integrating lateral relationships into the modeling framework.

The Fortran-based Schlumberger array^68^ code was employed for the 2D inversion of DC-VES data collected along two profiles. This algorithm interprets all DC-VES data collectively through a unified conductivity model, ensuring consistency in interpretation. Initially, a forward calculation is conducted at each VES data point using a homogeneous earth model with a resistivity of 30 Ω m. Notably, the models incorporate topographic effects, enhancing their accuracy and relevance for the studied region. A flowchart of this algorithm is illustrated as shown in Fig. 4b.

Google Earth Pro was used to obtain satellite images of the study area (Version:7.3, URL link: https://www.google.com/earth/about/versions/). Surfer (Version:13.6, URL link: www.goldensoftware.com) and Matlaab (Version: 9.3, URL link: https://www.mathworks.com/) were used to generate the maps and cross sections resulting from the above inversion results, respectively.

### Model setup of constrained inversion techniques

Figure 4a illustrates the model setup for inversion techniques that incorporate vertical, lateral, and spatial constraints between adjacent models along a profile. When the coupling width is set to one (CW = 1), constraints are applied solely in the vertical direction, as there is no linkage between neighboring models. These vertical constraints connect basic parameters such as resistivity and thickness within the same model, with the exception of the final layer, which remains unconstrained^67^. In this study, the models consist of six layers, where the resistivity of each layer is sequentially constrained by the resistivity of the subsequent layer, establishing a dependent relationship from the first to the sixth layer.

Lateral constraints denote the interconnection between adjacent models, wherein the output parameters of one model are laterally coupled to those of the subsequent model in a sequential manner. When a model is constrained by its adjacent counterpart, a coupling width of two (CW = 2) is applied, encompassing one vertical constraint on the model itself and one lateral constraint linking it to the neighboring model. This approach is integral to the SCI technique utilized in analyzing DC resistivity data^67^. The interdependencies between these constraints ensure a cohesive and systematic modeling process.

### Basic theory of LCI and SCI inversions

The LCI method shares a similar mathematical framework with the SCI method^19,20^, both employing least-squares inversion techniques to model a regular stratified Earth structure while incorporating spatial constraints to ensure smooth lateral transitions (Fig. 4a; flow chart of Aarhus algorithm^23^). The inversion problem is expressed either as Eq. (1) or its compact form, Eq. (2). Here, the Jacobian matrix (G) represents the partial derivatives mapping the ath data point and the bth model parameter, encapsulating data sensitivity. The roughening matrix (R) encapsulates lateral or spatial constraints, while the vector (δd_obs_) represents the discrepancy between observed data and the reference model. The error in the observed data is denoted by the vector (e_obs_), and the constraints are expressed through the vector (δr). Additionally, (e_r_) signifies the error associated with these constraints, which is expected to average zero^17,19,20^.

1 $$\left[ {\begin{array}{*{20}c} G \\ R \\ \end{array} } \right].\delta m_{true} = \left[ {\begin{array}{*{20}c} {\delta d_{obs} } \\ {\delta r} \\ \end{array} } \right] + \left[ {\begin{array}{*{20}c} {e_{obs} } \\ {e_{r} } \\ \end{array} } \right]$$

2 $$G^{\prime } .\delta m_{true} = \delta d^{\prime } + e^{\prime }$$

The distinction between LCI and SCI hinges on the structure of the R matrix, which defines parameter constraints. In LCI, the R matrix (Eq. 3) establishes connections solely along VES profiles using binary values (1 and -1) for constrained parameters. Conversely, SCI’s R matrix (Eq. 4) extends these links both along and across profiles^20^, incorporating a broader constraint network. Here, Nj denotes the number of models associated with the jth parameter, while the summation of -1 in the jth row equals Nj^19^.

3 $$R = \left[ {\begin{array}{*{20}l} 1 \hfill & 0 \hfill & \cdots \hfill & 0 \hfill & { - 1} \hfill & 0 \hfill & \cdots \hfill & 0 \hfill & 0 \hfill & 0 \hfill \\ 0 \hfill & 1 \hfill & 0 \hfill & \cdots \hfill & 0 \hfill & { - 1} \hfill & 0 \hfill & \cdots \hfill & 0 \hfill & 0 \hfill \\ \vdots \hfill & \vdots \hfill & \vdots \hfill & \vdots \hfill & \vdots \hfill & \vdots \hfill & \vdots \hfill & \vdots \hfill & \vdots \hfill & \vdots \hfill \\ 0 \hfill & 0 \hfill & 0 \hfill & \cdots \hfill & 0 \hfill & 1 \hfill & 0 \hfill & \cdots \hfill & 0 \hfill & { - 1} \hfill \\ \end{array} } \right]$$

4 $$R = \left[ {\begin{array}{*{20}l} {N^{1} } \hfill & 0 \hfill & \cdots \hfill & 0 \hfill & { - 1} \hfill & 0 \hfill & \cdots \hfill & 0 \hfill & { - 1} \hfill & 0 \hfill & \cdots \hfill & 0 \hfill & 0 \hfill & 0 \hfill \\ 0 \hfill & {N^{2} } \hfill & 0 \hfill & \cdots \hfill & 0 \hfill & { - 1} \hfill & 0 \hfill & \ldots \hfill & 0 \hfill & { - 1} \hfill & 0 \hfill & \cdots \hfill & 0 \hfill & 0 \hfill \\ \vdots \hfill & \vdots \hfill & \vdots \hfill & \vdots \hfill & \vdots \hfill & \vdots \hfill & \vdots \hfill & \vdots \hfill & \vdots \hfill & \vdots \hfill & \vdots \hfill & \vdots \hfill & \vdots \hfill & \vdots \hfill \\ 0 \hfill & 0 \hfill & 0 \hfill & \cdots \hfill & 0 \hfill & {N^{j} } \hfill & 0 \hfill & \ldots \hfill & 0 \hfill & { - 1} \hfill & 0 \hfill & \cdots \hfill & 0 \hfill & { - 1} \hfill \\ \end{array} } \right]$$

Equation (5) defines the objective function (Q), incorporating (ND) (the number of data points), (NC) (the number of constraints), and (C′) (the covariance matrix of joint observation error (e)). Minimization of (Q) is achieved through Eq. (6), which optimally balances data mismatch and model roughness, as dictated by the constraints^19^.

5 $$Q = \left( {\frac{1}{ND + NC}\left[ {\delta d^{\prime T} C^{\prime - 1} \delta d^{\prime } } \right]} \right)^{1/2}$$

6 $$\delta m_{est} = \left( {G^{\prime T} .C^{\prime - 1} .G^{\prime} } \right)^{ - 1} G^{\prime T} C^{\prime - 1} \delta d^{\prime }$$

### Constrains and quality control on model parameters

A priori information is transferred between adjacent soundings via constraints imposed on model parameters, including resistivity, thickness, and depth, to account for anticipated local geological variations^17,19^. These constraints are expressed as relative values exceeding zero, with a value of 0.1 indicating a binding uncertainty of approximately 10%. Parameters lacking constraints are denoted by either extremely high values or a negative value of -1, signifying their unconstrained status^17,19,20^.

The inversion process integrates both data and constraints, necessitating a balanced approach to produce reliable model parameters. Parameters with minimal impact on the data are predominantly governed by constraints, while those significantly influencing the data rely more heavily on observational inputs^17^. This interdependency facilitates the transfer of parameter information between soundings, enabling well-resolved regions to inform poorly resolved ones via constraint propagation, thereby enhancing overall model accuracy and coherence.

Inverting model segments from the LCI often complicates distinguishing between data-driven and constraint-influenced components. Geological variability in the area informs constraints that enhance inversion processes^16,25^, providing valuable inputs while ensuring consistency with local survey data. These constraints guide neighboring soundings, offering recommendations that align with regional geological expectations without compromising data fit, thereby improving the overall accuracy and reliability of inversion outcomes.

The quality control of constrained inversion models is achieved through a comprehensive sensitivity analysis of the final model parameters derived from both LCI and SCI inversions. This sensitivity is approximated linearly using the R matrix of the estimation error (C_est_)^20^. The standard deviation factor (STDF), defined in Eq. 7, serves as a critical tool for evaluating parameter resolution. An STDF value of one signifies perfect resolution, while values less than 1.3, 1.5 and 2 indicate well-resolved, moderately resolved, and poorly resolved parameters, respectively. Parameters with an STDF exceeding two are deemed unresolved^20^. The SCI approach enhances parameter resolution by incorporating information in two dimensions along and across profile lines, enabling better resolution of poorly defined parameters, particularly in noisy environments.

7 $$STDF\left( {m_{s} } \right) = exp\left( {C_{est,ss}^{1/2} } \right)$$

### Validation method using ROC-AUC curve

The receiver operating characteristic (ROC) curve serves as a critical tool for validating maps of potential groundwater areas generated through methods like the analytical hierarchy process (AHP). It evaluates the performance of multi-class classification problems by illustrating the trade-off between the false positive rate (FPR) and the true positive rate (TPR). The area under the ROC curve (AUC) quantifies the model’s predictive accuracy, with higher values indicating better classification performance^69–71^. This metric has been widely employed in various fields, including groundwater potential mapping, geological hazard assessment, and rockfall susceptibility analysis^55,72,73^*.*

Mathematically, the ROC curve evaluates a model’s discriminative power by plotting its sensitivity (TPR) against 1-specificity (FRP), with the AUC (area under the curve) quantifying overall performance. The ROC curve is constructed using Eqs. (8 and 9) derived from true positives (TP), true negatives (TN), false positives (FP), and false negatives (FN), which represent the proportions of correctly and incorrectly classified pixels. TP and TN represent the accurate classification of positive and negative pixels, respectively, whereas FP and FN denote incorrect classifications.

8 $$Sensitivity = TP/\left( {TP + FN} \right)$$

9 $$Specificity = TN/\left( {TN + FP} \right)$$

The AUC accuracy spectrum ranges from 0 to 1, with 1 denoting perfect alignment between predictions and reference data. A ROC value of 0 indicates no discrepancy between groundwater potential and reference data, signifying perfect alignment. An AUC of 0.5 represents no discriminatory capability, equivalent to random guessing, while values below 0.5 indicate performance worse than chance. Conversely, a high ROC value demonstrates significant differentiation, thereby reflecting enhanced model efficacy. An ideal model’s ROC curve should closely hug the upper left corner, reflecting optimal sensitivity and specificity. Conversely, a model indistinguishable from random guessing will exhibit an ROC curve aligned diagonally at a 45-degree angle, signifying equal true-positive and false-positive rates.

Models with AUCs between 0.7 and 0.8 are deemed acceptable, those between 0.8 and 0.9 are considered excellent, and scores exceeding 0.9 reflect outstanding performance. Thus, the ROC curve and AUC provide a robust framework for assessing model effectiveness in distinguishing positive and negative classes.

## Result and discussions

### Analysis of conventional datasets

**Fig. 5:**
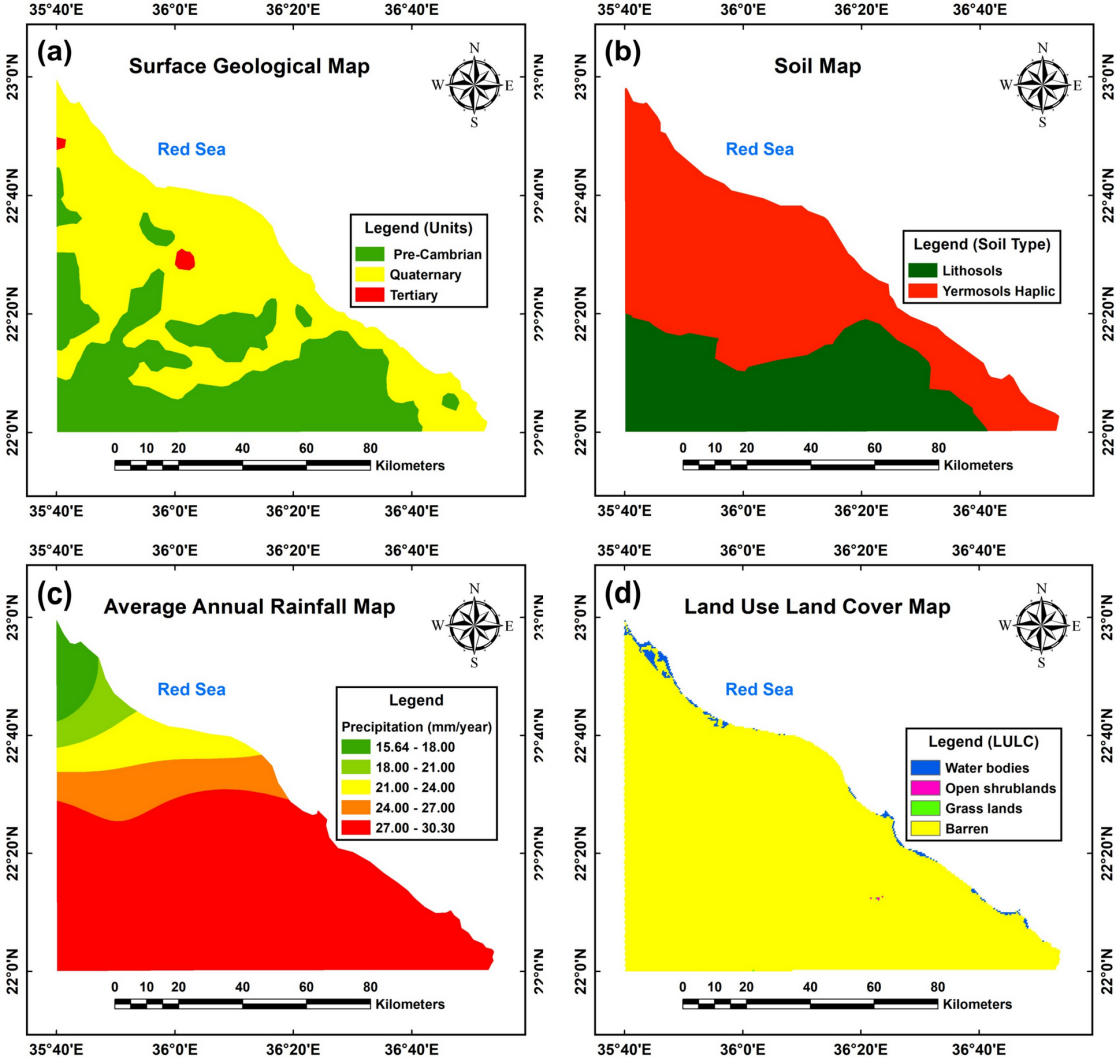
Surface geological map (**a**), digital soil map (**b**), land use land cover (LULC) map (**c**) and annual rainfall map (**d**) of the study area. (The maps shown in the figure were obtained and compiled using ArcGIS 10.5 (https://www.esri.com/) and Surfer 13.6 (www.goldensoftware.com), respectively, by Mohamed Genedi).

The surface geological map (Fig. 5a) reveals three primary rock units in the study area: Quaternary (yellow zone), Tertiary (red zone), and Precambrian basement rocks (green zone). Quaternary deposits dominate, particularly along the western, northern, and northeastern coastal regions, while Precambrian mafic and ultramafic rocks are prevalent in the southern, southwestern, and western sections. Tertiary rocks, meanwhile, occupy restricted areas in the central and northwestern parts of the region. These units collectively define the area’s geological framework, highlighting their spatial interdependencies and distinct distribution patterns.

The digital soil map (Fig. 5b) categorizes the region into two primary soil types: lithosols and Yermosol-Hapalik. Lithosols, represented by the dark green zone in the southern and southwestern areas, are characterized by shallow depths due to underlying hard rocks within 10 cm of the surface. In contrast, Yermosol-Hapalik, depicted in red and covering the remaining areas, exhibits a dry moisture regime without permafrost within 200 cm of the surface^38^.

Figure 5c presents the spatial interpolation map of rainfall in the study area, with annual precipitation for 2021–2022 ranging from 15.64 to 30.30 mm/year. This data was categorized into five classes: very low (15.64–18.00 mm/yr), low (18.00–21.00 mm/yr), medium (21.00–24.00 mm/yr), high (24.00–27.00 mm/yr), and very high (27.00–30.30 mm/yr). The distribution reveals a southward increase in rainfall, indicating very high groundwater potential, while the northern region exhibits a decline due to low humidity, high temperatures, and evaporation, classifying it as having very low groundwater potential.

### Results of remote sensing datasets

#### Analysis of Esri Sentinel-2 LULC data

The study area, depicted in Fig. 5d, is predominantly characterized by barren lands (yellow zone), with limited occurrences of water bodies (blue zone) along the Red Sea coast, open shrublands (pink zone), and grasslands (green zone). Barren lands, primarily consisting of desert and highland regions, dominate the study area, where ecosystems are largely defined by soil prevalence and minimal vegetation. Urban expansion’s specific impacts remain unidentified in this context, as the region’s permeable surfaces, coupled with high infiltration rates and low runoff, suggest enhanced groundwater recharge potential.

#### Analysis of ASTER-GDEM data

**Fig. 6:**
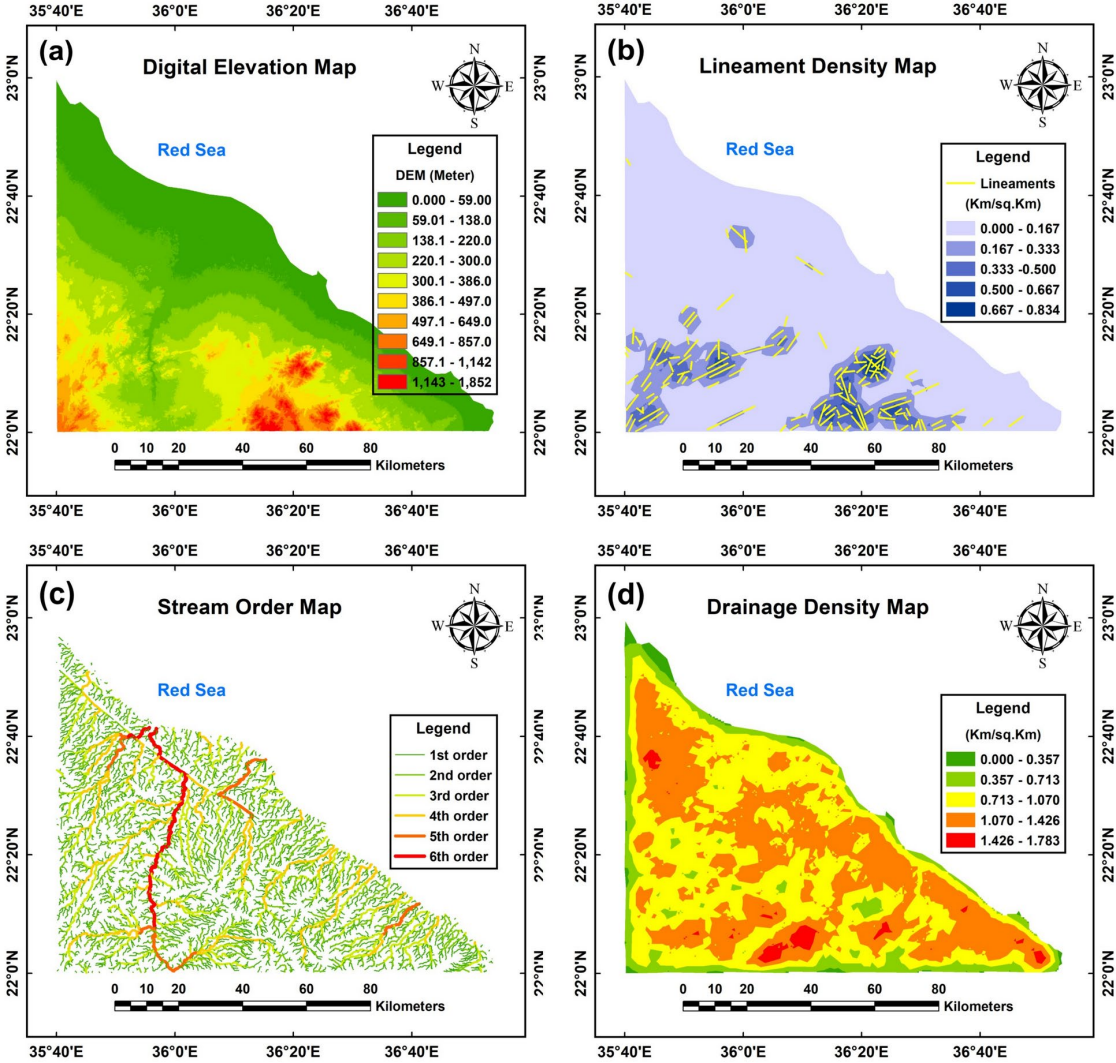
The hydrogeologic analysis from ASTER-GDEM raster data. Digital elevation map (**a**), lineament density map (**b**), stream order map (**c**) and drainage density map (**d**) of the study area. (The maps shown in the figure were obtained and compiled using ArcGIS 10.5 (https://www.esri.com/) and Surfer 13.6 (www.goldensoftware.com), respectively, by Mohamed Genedi and Noura Gouhar).

The study area (Fig. 6a) exhibits elevation values ranging from 0 to 1852 m above sea level, categorized into ten distinct classes; 0–59 m, 59.01–138 m, 138.1–220 m, 220.1–300 m, 300.1–386 m, 386.1–497 m, 497.1–649 m, 649.1–857 m, 857.1–1142 m, and 1143–1852 m. These classes incrementally delineate the terrain, with the highest elevations (red zone) concentrated in the southern and southwestern regions, transitioning to lower elevations (green zone) toward the northern and northeastern coastal areas. The prioritization process emphasized high-elevation areas as the primary focus, with moderate-elevation regions and plains receiving subsequent consideration.

#### Hydrogeological analysis of ASTER-GDEM data

The lineament density map (Fig. 6b) was derived from the Hillshade map by applying standardized azimuth and altitude values to determine lineament orientation and spatial distribution. These data were subsequently categorized into five classes: very low (0.000–0.167 km/km^2^, green), low (0.167–0.333 km/km^2^, light green), moderate (0.333–0.500 km/km^2^, yellow), high (0.500–0.667 km/km^2^, orange), and very high (0.667–0.834 km/km^2^, red) density zones. Notably, the predominant density range of 0.000–0.167 km/km^2^, covering much of the study area, indicates limited groundwater potential. The southern and southwestern regions, characterized by higher and very high densities (0.500–0.8234 km/km^2^), predominantly consist of igneous rocks exhibiting fractures that contribute to secondary porosity. These area are categorized as possessing high and very high potential.

The waterway network map (Fig. 6c) categorizes the study area into six stream orders, reflecting a hierarchical classification based on waterway capacity. First-order streams, depicted as green lines, represent the smallest channels, while higher orders emerge where these converge, progressively increasing in size and capacity. The sixth-order stream, indicated by a bold red line flowing from south to north, signifies the main channel with the greatest water capacity, integrating all lower-order tributaries within the study area. The hierarchical organization of stream orders plays a critical role in shaping regional hydrological systems.

Figure 6d presents a drainage density map categorized into five classes: very low (0.000–0.357 km/km^2^, green), low (0.357–0.713 km/km^2^, light green), moderate (0.713–1.070 km/km^2^, yellow), high (1.070–1.426 km/km^2^, orange), and very high (1.426–1.783 km/km^2^, red). The majority of the region exhibits medium to high drainage density (0.713–1.426 km/km^2^), suggesting moderate to limited groundwater potential. Conversely, areas with very low to low drainage density (0.000–0.713 km/km^2^) along the periphery, as depicted in Fig. 6d, indicate higher groundwater potential. Notably, the southern regions, characterized by very high drainage density (1.426–1.783 km/km^2^), exhibit reduced groundwater potential due to geological factors such as igneous rock formations and steep slopes that hinder rainfall infiltration.

#### Topographic analysis of ASTER-GDEM data

**Fig. 7:**
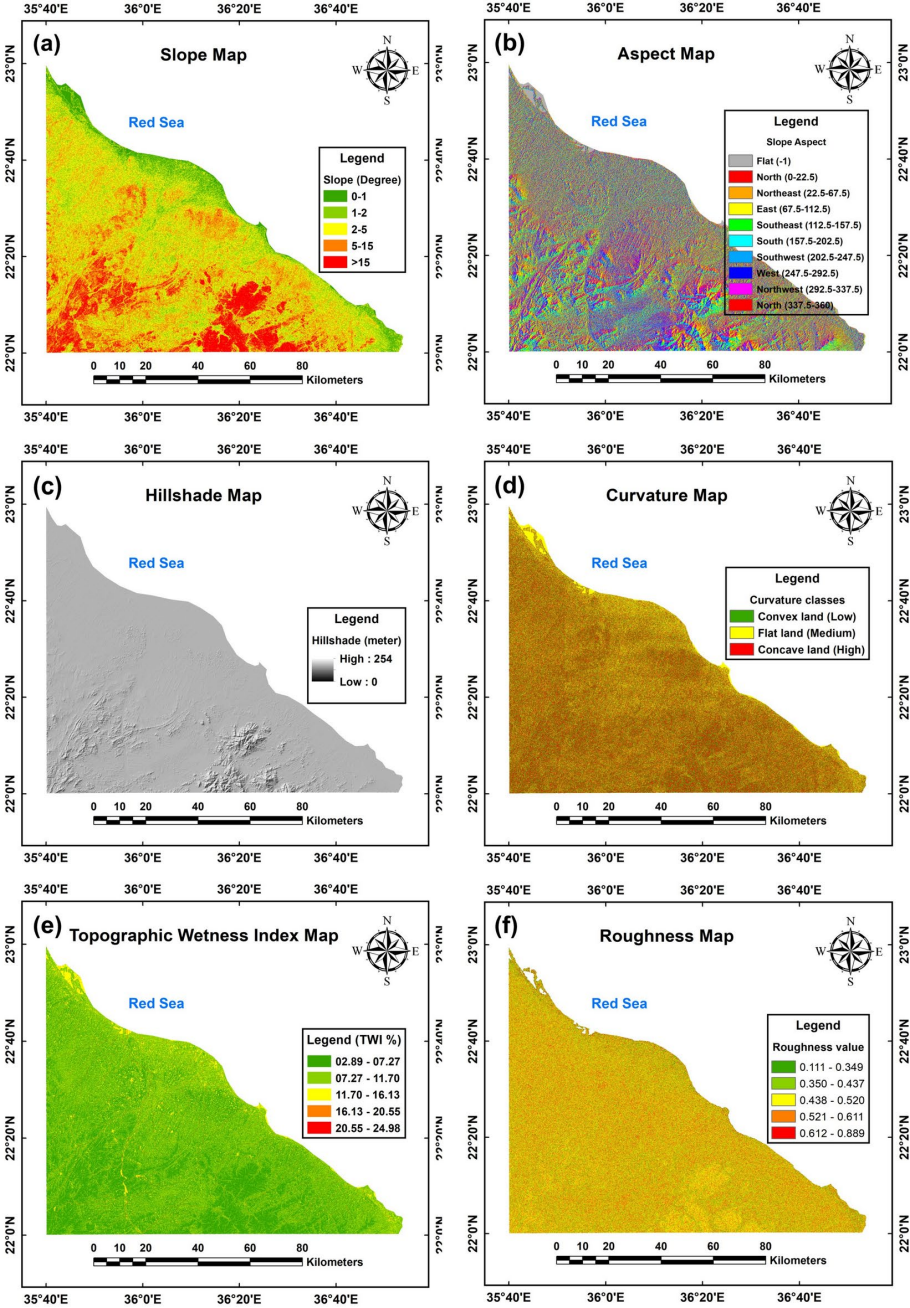
The topographic analysis from ASTER-GDEM raster data. Slope map (**a**), aspect map (**b**), hillshade map (**c**), curvature map (**d**), topographic wetness index (TWI) map (**e**) and roughness map (**f**) of the study area. (The maps shown in the figure were obtained and compiled using ArcGIS 10.5 (https://www.esri.com/) and Surfer 13.6 (www.goldensoftware.com), respectively, by Mohamed Genedi and Noura Gouhar).

The study area, as depicted in Fig. 7a, exhibits a slope gradient ranging from 0 to 66.46 degrees, categorized into five distinct classes. These include flat areas (0–1 degrees, dark green), nearly level areas (1–2 degrees, green), gentle slopes (2–5 degrees, yellow), moderate slopes (5–15 degrees, orange), and steep to very steep slopes (15–66.46 degrees, red). Notably, the region prioritizes areas with slopes ranging from almost flat to moderate (dark green to orange zones), whereas the southern and southwestern sections are characterized by steep to very steep gradients (red zone).

The aspect slope of the region depicted in Fig. 7b is categorized into ten classes: no aspect direction for flat areas (− 1), and eight directional divisions ranging from north (0–22.5) to northwest (292.5–337.5) with north-directed slopes concluding the classification (337.5–360). The southern and southwestern sections exhibit diverse shading due to varying slope orientations, while the northern and northeastern coastal areas are predominantly flat, represented by a gray zone.

Figure 7c presents a hillshade map of the study area, utilizing standard azimuth and altitude values of 315° and 45°, respectively. The northern, northeastern, and central regions exhibit a relatively flat topography, indicated by high hillshade values (light gray zones), while the southern and southwestern sections display progressively darker gray tones, signifying elevated mountainous terrain.

The land surface curvature in this region (Fig. 7d) spans from − 12.80 to 13.76 and is categorized into three zones: convex (− 12.80 to 0.1), flat (− 0.1 to 0.1), and concave (0.1 to 13.76). Concave lands, indicated by positive curvature values, are associated with water accumulation and are assigned higher weights, reflecting their significance, while lower weights correspond to lesser curvature values.

The Topographic Wetness Index (TWI) for the study area (Fig. 7e) spans a range of 2.89 to 24.98, reclassified into five categories: 2.89–7.27, 7.27–11.7, 11.7–16.13, 16.13–20.55, and 20.55–24.98. Values between 2.89 and 11.7, represented by dark and light green zones, dominate the southern and southwestern regions, aligning with the irregular topography characteristic of areas classified as having very low to low groundwater potential. Conversely, medium to high TWI values (yellow and orange zones) occur in specific areas with more regular topography, indicating medium to high groundwater potential. Higher weights were assigned to greater TWI values, reflecting their stronger association with groundwater presence.

Figure 7f presents the roughness map of the study area, with values spanning from 0.111 to 0.889, which were categorized into five classes: 0.111–0.349, 0.350–0.437, 0.438–0.520, 0.521–0.611, and 0.612–0.889. Notably, higher values signify rougher surfaces, whereas lower values represent smoother ones, illustrating a direct correlation between numerical magnitude and surface texture.

#### Analysis of ASTER-L1B data

**Fig. 8:**
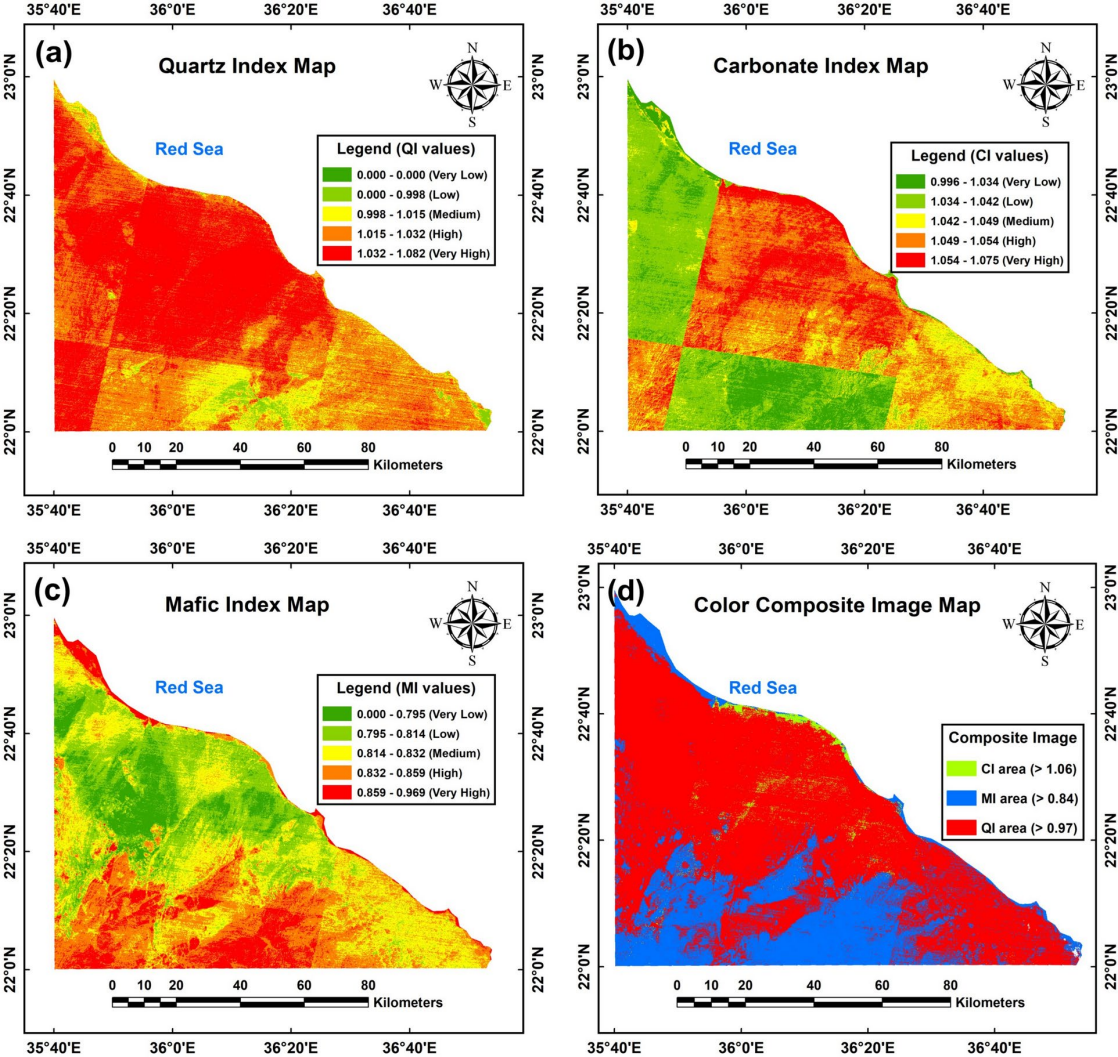
The classifications of mineral indexes from ASTER-L1B raster data. Quartz index (QI) map (**a**), carbonate index (CI) map (**b**), mafic index (MI) map (**c**), and color composite image map (**d**) of these mineral indexes together in this area. (The maps shown in the figure were processed, obtained and compiled using ENVI 5.3 (https://www.nv5geospatialsoftware.com/), ArcGIS 10.5 (https://www.esri.com/) and Surfer 13.6 (www.goldensoftware.com), respectively, by Mohamed Genedi).

Figure 8a–c presents ASTER-TIR images depicting the QI, CI, and MI indices, with each index displayed in Fig. 8a–c, respectively. These images have undergone linear contrast stretching, ranging from 0 to 1.082 for QI, 0.996 to 1.075 for CI, and 0 to 0.969 for MI, enhancing their interpretability. The integration of these processed images is crucial for detailed geological analysis and satellite-based interpretation of the study area, facilitating the identification of rock types and elucidating the spatial distribution of geological outcrops.

The study area has been categorized into five distinct zones based on Quality Index (QI) and Carbonate Index (CI), as depicted in Fig. 8a, b*.* QI zones range from very low (dark green, QI = 0) to very high (red, QI = 1.032–1.082), with the north-central, north-western, and south-western regions predominantly exhibiting high to very high QI values, while the southern and south-eastern areas show medium to very low values. Similarly, CI zones are classified from very low (dark green, CI = 0.996–1.034) to very high (red, CI = 1.054–1.075). The north-western, western, and southern regions exhibit low to very low CI values (dark green to green zones), contrasting with medium to very high CI values in other areas. Notably, the very high CI values are predominantly concentrated in the north-central part of the region.

Figure 8c presents the MI map, categorized into five classes: very low (MI < 0.795, dark green), low (MI = 0.795–0.814, light green), moderate (MI = 0.814–0.832, yellow), high (MI = 0.832–0.859, orange), and very high (MI = 0.859–0.969, red). Notably, the north-central areas are characterized by low to very low MI values, while high to very high MI values dominate the southern and southwestern sections. Intermediate MI values are distributed across the remaining regions.

Figure 8d presents a color composite image (CCI) map derived from ASTER-TIR data, illustrating quartz (QI), carbonate (CI), and mafic (MI) indices. Pixels with QI > 0.97, indicative of strong quartz signals, are highlighted in yellow, while those exceeding CI > 1.06, representing high carbonate content, appear in green. Additionally, areas with MI > 0.84, denoting significant mafic mineral presence, are marked in red. These thresholds define distinct zones based on spectral characteristics.

The geological characteristics of Area A suggest a dominance of quartz-rich sedimentary rocks, as indicated by the moderate to very high QI, very low to very high CI, and moderate to very low MI values, represented by red zones in Fig. 8d. Notably, this region is superficially overlain by Quaternary rock units, delineated by yellow zones in the surface geological map (Fig. 5a). The CCI image (area B) reveals limited light green zones characterized by low to very high QI, very high CI, and low to very low MI. These patterns are attributable to the prevalence of sedimentary rocks, particularly limestone, which contains carbonate minerals like calcite and dolomite that significantly influence CI values. Area (C), situated in the southern region of the CCI image (highlighted in blue in Fig. 8d), exhibits low QI and CI values alongside high to very high MI values. These characteristics suggest the presence of mafic or ultramafic igneous rocks, a hypothesis corroborated by the surface geological map (Fig. 5a), which indicates Precambrian rock units in the southern, southwestern, and western sectors. The southwestern regions (D) of the CCI image (Fig. 8d) exhibit complex red and yellow colorations, suggesting a mixture of materials. These may include igneous rocks with high MI, potentially overlain by unconsolidated sedimentary rocks characterized by high QI and CI (Fig. 8a, b).

The CCI image delineates distinct geological features through color-coded zones, revealing spatial distributions and characteristics of rock types. Yellow zones signify quartz-rich sedimentary rocks in the northeastern, north-central, and western regions, characterized by pixel values exceeding 0.97. Light green zones highlight carbonate-rich sedimentary rocks along the central and northern Red Sea coast in limited parts, with pixel values above 1.06. Both quartz- and carbonate-rich formations exhibit high permeability, making them key groundwater sources. Conversely, red zones denote mafic and ultramafic igneous rocks in the southern and southwestern areas, marked by pixel values greater than 0.84. The correlation between these image-derived insights and existing geological maps underscores the reliability of the remote sensing analysis.

The basement rocks in the southern and southwestern sections of the study area exhibit high MI due to the prevalence of ultramafic and mafic compositions (Fig. 8c). Conversely, MI decreases progressively as the rock type transitions to quartz-rich formations in the north-central region. Mafic terrains generally exhibit low permeability and productivity, rendering them ineffective as groundwater aquifers unless fractured.

#### Analysis of AHP method

Nine thematic layers, generated in point raster format through remote sensing and conventional datasets (e.g., Quartz Index, Carbonate Index, Mafic Index, Slope, Lineament Density, Drainage Density, rainfall, Soil, and Land Use/Land Cover parameters), were analyzed to assess their influence on groundwater flow and storage. As presented in Tables 2 and 3, the PCM and NPCM matrices ranked these layers in descending order of significance. Their associations were weighted based on their relevance to groundwater occurrence, researcher’s opinion with incorporating expert judgment. Higher weights signify greater impact on groundwater potential, while lower weights indicate lesser influence. Subcategories within each layer were reclassified using the natural breaks classification method in GIS, assigning ranks from one to five to denote very low to very high impacts on groundwater potential, respectively.

The Quartz Index (QI) emerged as the most significant factor influencing groundwater potential, followed by the Carbonate Index (CI), slope, rainfall, drainage density (DD), topographic wetness index (TWI), lineament density (LD), and land use/land cover (LULC) coefficients. The Mafic Index (MI) was deemed the least influential due to the low porosity and permeability of mafic rocks. QI and CI were prioritized because quartz and carbonate-rich rocks exhibit high porosity and permeability, facilitating groundwater movement. Slope ranked third in importance, as the predominantly flat or gentle terrain enhances water infiltration while minimizing surface runoff.

The normalized weights assigned to these factors were 0.2816 (QI), 0.2098 (CI), 0.1806 (slope), 0.1354 (rainfall), 0.0705 (DD), 0.0492 (TWI), 0.0335 (LD), 0.0217 (LULC), and 0.0178 (MI). The consistency index (CI) and highest eigenvalue (λ_max_) were calculated as 0.1429 and 9.6273, respectively, yielding a consistency ratio (CR) of 0.0959 based on a random index (RI) of 1.32 for nine thematic layers. Since CR < 0.1, the pairwise comparison matrix (PCM) demonstrated acceptable consistency^59^. Further details, including parameter descriptions, normalized weights derived via the AHP method, and class rankings, are outlined in Table 5.

**Table 5:** Illustrates the normalized weights and assigned ranks of thematic layers.

Parameter	Unit	Normalized Weight	Influence (%)	Classes		Rank
Quartz Index (QI)		0.28158	28.16	0	Very low QI	1 (Very low rank)
			28.16	(0.000–0.998)	Low QI	2 (Low rank)
			28.16	(0.998–1.015)	Medium QI	3 (Medium rank)
			28.16	(1.015–1.032)	High QI	4 (High rank)
			28.16	(1.032–1.082)	Very high QI	5 (Very high rank)
Carbonate Index (CI)		0.20978	20.98	(0.996–1.034)	Very low CI	1 (Very low rank)
			20.98	(1.034–1.042)	Low CI	2 (Low rank)
			20.98	(1.042–1.049)	Medium CI	3 (Medium rank)
			20.98	(1.049–1.054)	High CI	4 (High rank)
			20.98	(1.054–1.075)	Very high CI	5 (Very high rank)
Slope	(Degree)	0.18061	18.06	(0–1)°	Flat	5 (Very high rank)
			18.06	(1–2)°	Nearly level	4 (High rank)
			18.06	(2–5)°	Gentle	3 (Medium rank)
			18.06	(5–15)°	Moderate	2 (Low rank)
			18.06	(> 15)°	Steep to very steep	1 (Very low rank)
Rainfall	(mm/year)	0.13536	13.54	(15.64–18.00)	Very low rainfall	1 (Very low rank)
			13.54	(18.00–21.00)	Low rainfall	2 (Low rank)
			13.54	(21.00–24.00)	Medium rainfall	3 (Medium rank)
			13.54	(24.00–27.00)	High rainfall	4 (High rank)
			13.54	(27.00–30.30)	Very high rainfall	5 (Very high rank)
Drainage Density (DD)	(Km/sq.Km)	0.07050	7.05	(0.000–0.357)	Very low	5 (Very high rank)
			7.05	(0.357–0.713)	Low	4 (High rank)
			7.05	(0.713–1.070)	Medium	3 (Medium rank)
			7.05	(1.070–1.426)	High	2 (Low rank)
			7.05	(1.426–1.783)	Very high	1 (Very low rank)
Topographic Witness Index (TWI)	(Percent, %)	0.04920	4.92	(02.89–07.27)	Very low TWI	1 (Very low rank)
			4.92	(07.27–11.70)	Low TWI	2 (Low rank)
			4.92	(11.70–16.13)	Medium TWI	3 (Medium rank)
			4.92	(16.13–20.55)	High TWI	4 (High rank)
			4.92	(20.55–24.98)	Very high TWI	5 (Very high rank)
Lineament Density (LD)	(Km/sq.Km)	0.03350	3.35	(0.000–0.167)	Very low density	1 (Very low rank)
			3.35	(0.167–0.333)	Low density	2 (Low rank)
			3.35	(0.333–0.500)	Medium density	3 (Medium rank)
			3.35	(0.500–0.667)	High density	4 (High rank)
			3.35	(0.667–0.834)	Very High density	5 (Very high rank)
Land use land Cover (LULC)		0.02170	2.17	Water bodies	High GWPZ	4 (High rank)
			2.17	Open shrublands	Very high GWPZ	5 (Very high rank)
			2.17	Grass lands	Very high GWPZ	5 (Very high rank)
			2.17	Barren area	Very low GWPZ	1 (Very low rank)
Mafic Index (MI)		0.01778	1.78	(0.000–0.795)	Very low MI	5 (Very high rank)
			1.78	(0.795–0.814)	Low MI	4 (High rank)
			1.78	(0.814–0.832)	Medium MI	3 (Medium rank)
			1.78	(0.832–0.859)	High MI	2 (Low rank)
			1.78	(0.859–0.969)	Very high MI	1 (Very low rank)

The analytical hierarchy process (AHP) technique has been validated for groundwater potential assessment in arid regions, as demonstrated in prior studies conducted in similar environments^14,74^, including the Howden Valley near the current study area^14^. While the current study (see Table 5) and the previous research^14^ share comparable parameters, differences exist in the number of thematic layers and their assigned weights. Notably, there is close agreement in the weights of certain factors, such as lineament density (2.8% vs. 3.35%) and drainage density (5.3% vs. 7.05%), reflecting consistent contributions of these variables. However, discrepancies are observed in others, such as slope weight (29.6% vs. 18.06%), likely attributable to variations in topographic characteristics and slope angles between the study areas.

#### Groundwater potential zones (GWPZ) map

**Fig. 9:**
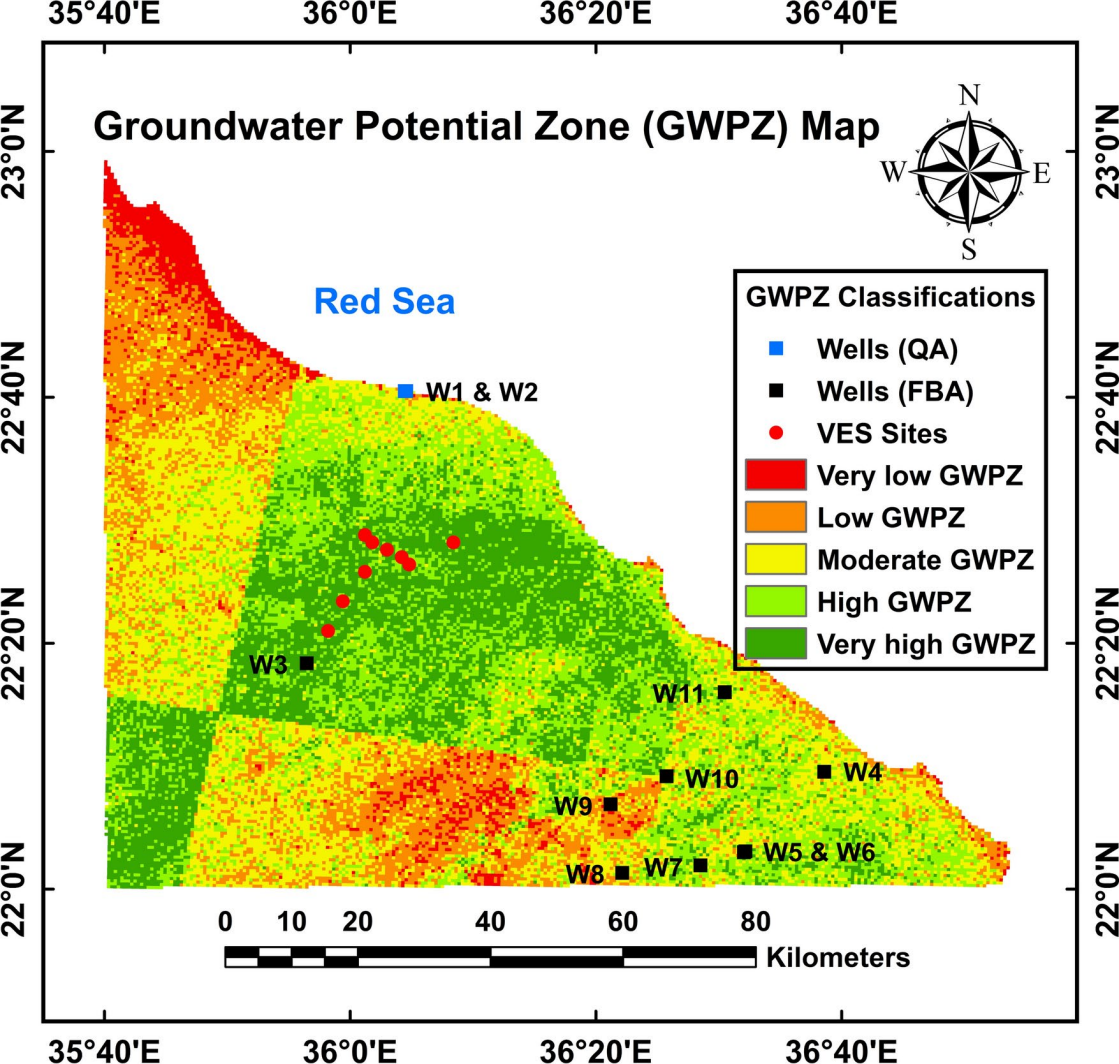
The classifications of the groundwater potential zones map of the investigated area where the red, orange, yellow, light green and dark green regions, reflect very low, low, moderate, high and very high zones, respectively. (The figure was obtained using ArcGIS 10.5 (https://www.esri.com/), by Mohamed Genedi).

The groundwater potential zone (GWPZ) map (Fig. 9), generated using ArcMap software through the AHP method, integrates nine layers via the raster calculator and weighted overlay techniques. This map categorizes the study area into five zones; very low, low, moderate, high, and very high potential zones comprising 4.53% (321.13 km^2^), 17.33% (1,229.56 km^2^), 27.05% (1,919.48 km^2^), 27.79% (1,971.42 km^2^), and 23.3% (1,653.3 km^2^), respectively, as summarized in Table 6.

**Table 6:** Groundwater potential zones classification and their area coverage.

Classification of Zones	Area (Km^2^)	Area (%)
Very Low GWPZ	321.13	4.53
Low GWPZ	1229.56	17.33
Moderate GWPZ	1919.48	27.05
High GWPZ	1971.42	27.79
Very High	1653.3	23.3

The region’s groundwater potential exhibits spatial variability, with low and very low potential areas (orange and red zones) predominantly located in the northwestern and southern parts. These zones correspond to mafic and ultramafic rock complexes characterized by low permeability and porosity, highlands, steep slopes, and high drainage density. Conversely, the north-central portion of the study area is classified as moderate to very high potential (yellow to dark green zones). These midlands feature quartz-rich deposits, Yermosols-type soils, medium-to-high rainfall, flat to gentle slopes (up to 15 degrees), and moderate to low drainage density. Notably, the main channels (5th and 6th stream order) are situated here, facilitating significant groundwater recharge. Consequently, these areas represent priority zones for groundwater well drilling due to their optimal hydrogeological characteristics.

### Inversion results of DC resistivity data

#### Non constrained and constrained inversion results

**Fig. 10:**
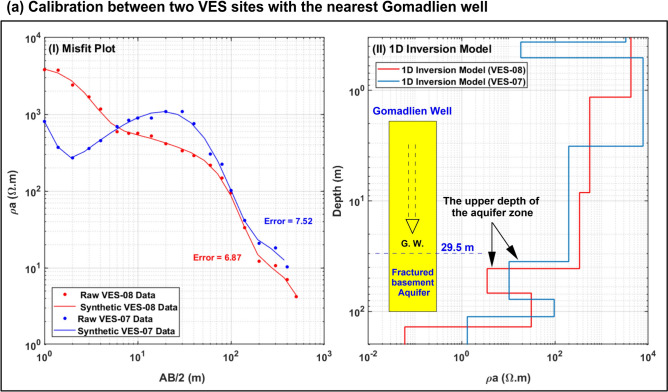
The calibration between VES-DC sites with the nearest neighbor Gomadlien well.(These plots were drawn using Matlaab 9.3 (https://www.mathworks.com/), and were collected using Surfer 13.6 (www.goldensoftware.com), respectively, by Mohamed Genedi and Gad El-Qady).

DC data were processed and inverted using the Aarhus algorithm, employing both unconstrained and constrained 1D inversion techniques with a uniform half-space resistivity of 30 Ω m as the starting model. Six geo-electrical layers were identified through VES-DC inversion, aligning with the four anticipated geological units in the study area. The observed and synthetic apparent resistivity data demonstrated strong agreement, and the results were calibrated against surface geological and well data. These findings were visualized as profiles using Matlab and Surfer, with red tones representing conductive structures and blue tones indicating resistive ones. During constrained inversion, six layers were selected based on three criteria: conventional 1D inversion outcomes, the number of geological units in the generalized profile, and sensitivity analysis of model parameters in LCI-DC and SCI-DC inversions. Figure 10 illustrates the calibration of 1D inversion results from VES-07 and VES-08 with data from the nearest borehole (Gomadien well)*.*

#### Non constrained inversion results

**Fig. 11.**
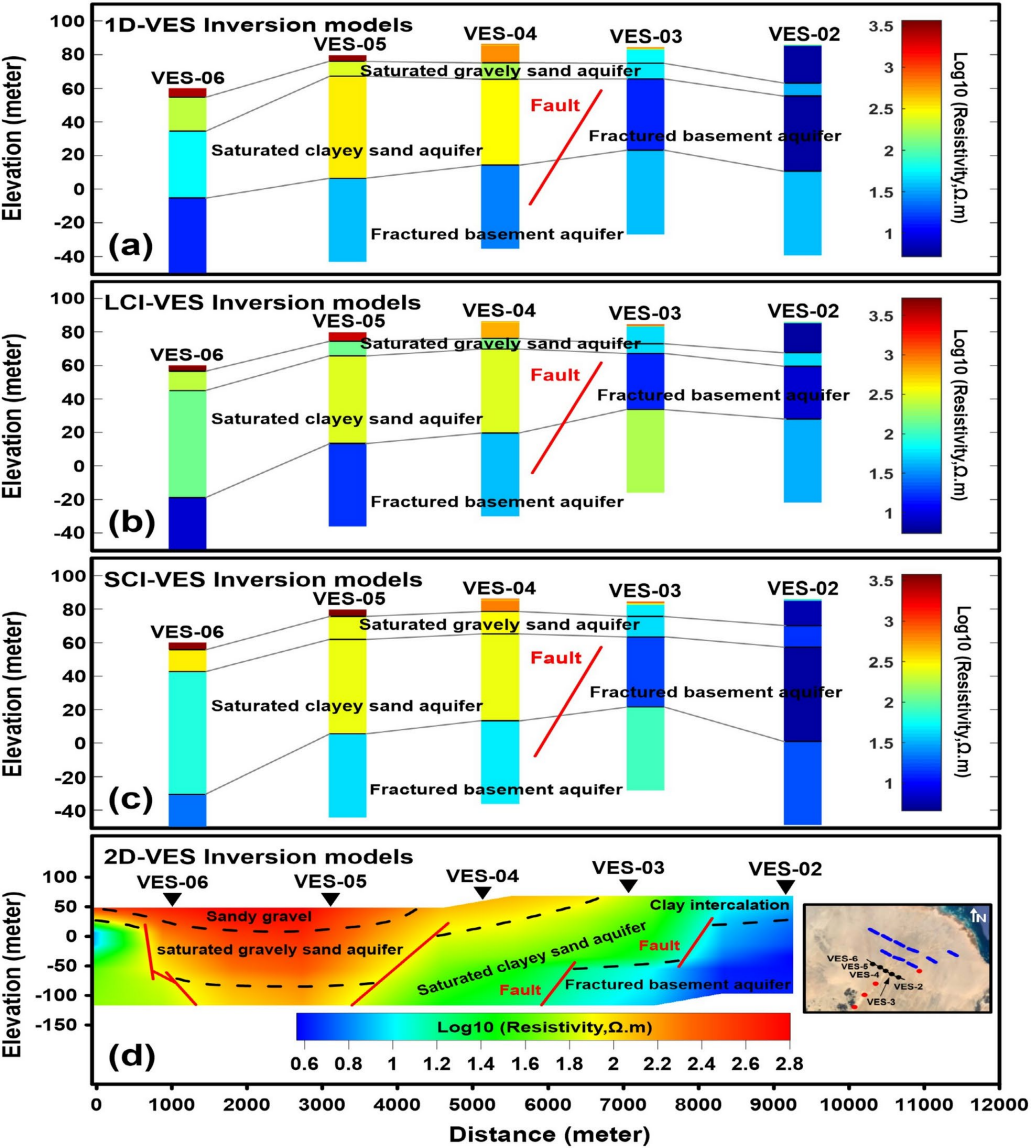
1D-VES non-constrained inversion model (**a**), lateral constrained inversion (LCI-VES) model (**b**), spatial constrained inversion (SCI-VES) model (**c**) and 2D-VES inversion model (**d**) of DC resistivity data along profile one. (The 1D-VES, LCI-VES and SCI-VES models were drawn using Matlaab 9.3 (https://www.mathworks.com/), while 2D-VES model was created using Surfer 13.6 (www.goldensoftware.com), respectively, by Mohamed Genedi).

**Fig. 12:**
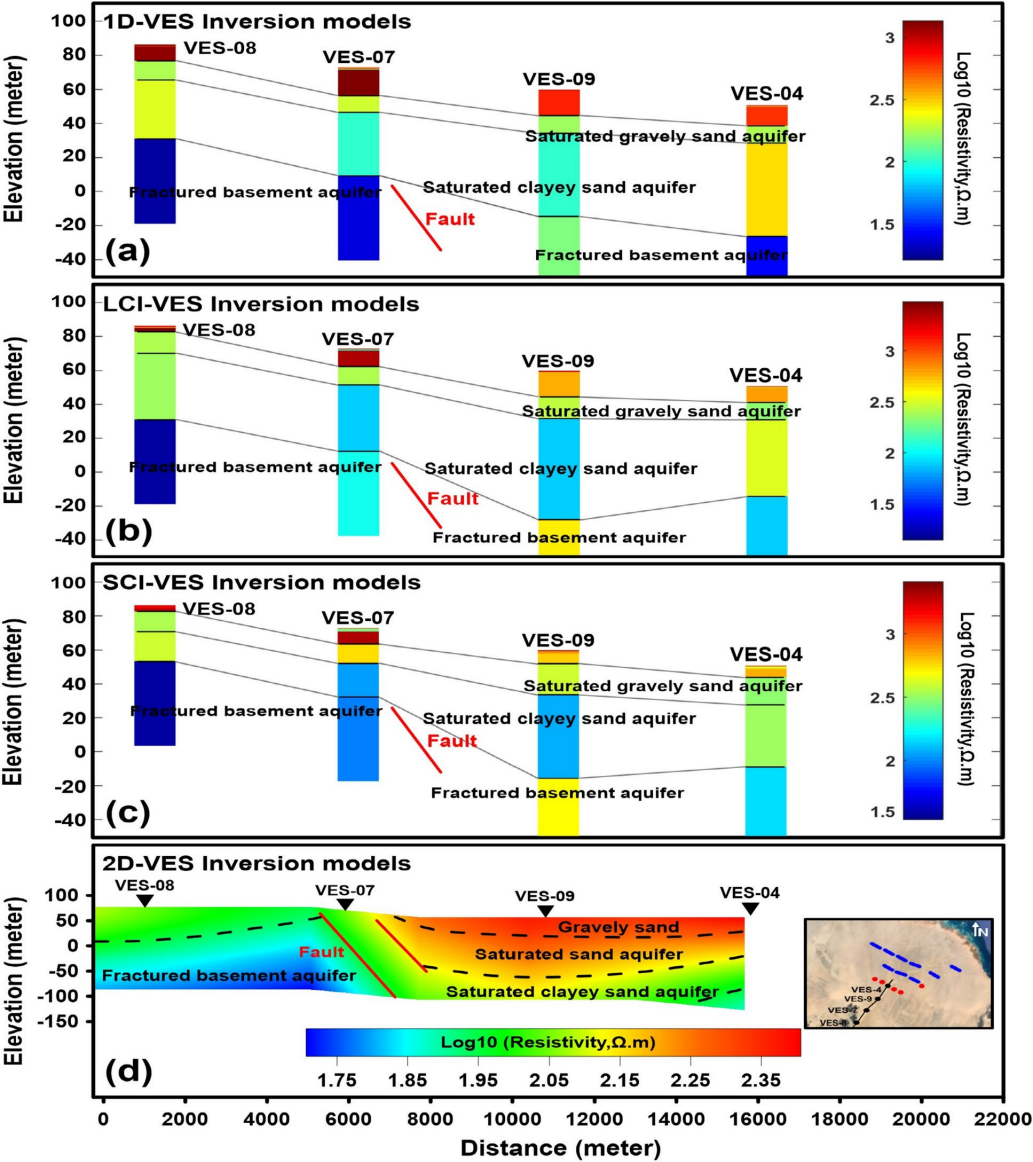
1D-VES non-constrained inversion model (**a**), lateral constrained inversion (LCI-VES) model (**b**), spatial constrained inversion (SCI-VES) model (**c**) and 2D-VES inversion model (**d**) of DC resistivity data along profile two. (The 1D-VES, LCI-VES and SCI-VES models were drawn using Matlaab 9.3 (https://www.mathworks.com/), while 2D-VES model was created using Surfer 13.6 (www.goldensoftware.com), respectively, by Mohamed Genedi).

The 1D-DC inversion models derived from measurements along two unrestricted profiles are presented in Figs. 11a and 12a. These models reveal six geoelectric layers characterized by resistivity values ranging from 5.15 to 3678 Ω m, corresponding to materials that include sand, sandy gravel, gravel, Pleistocene sediments, and fractured Precambrian basement rocks. The first layer exhibits variable resistivity (185.2–3678 Ω m) with a thickness of 0.28–0.94 m. The second layer shows resistivity values of 59.98–1878 Ω m, with a thickness of (0.11–0.59 m) and extending to depths of 0.28–0.94 m. The third layer displays resistivity variations of 5.48–3203 Ω m, spanning depths of 0.40–1.47 m and thicknesses of 3.40–22.26 m. The fourth layer is marked by high-to-low resistivity (31.6–221.4 Ω m), with a thickness of 7.21–19.94 m at depths of 3.96–23.14 m. The fifth layer features high-to-very-low resistivity (5.15–309.3 Ω m), reaching depths of 12.29–30.35 m and thicknesses of 34.31–60.62 m. Finally, the sixth layer exhibits medium-to-low resistivity (12.43–36.42 Ω m), except for a single high-resistivity value (138.4 Ω m) at VES-09, located at depths of 55.25–76.80 m.

#### Constrained inversion results

The DC electrical resistivity data were analyzed using LCI and SCI techniques, applying a constraint value of 0.3 to ensure correlation between the resistivity and thickness parameters of adjacent sites. A comprehensive sensitivity analysis was conducted on the final model parameters to validate the precision of the inverted, constrained models. The resulting models characterized by smooth transitions in both electrical resistivity and layer boundaries across the two profiles.

#### Lateral constrained inversion (LCI-VES) results

The LCI-DC models, depicted in Figs. 11b and 12b, incorporate lateral constraints across model parameters at adjacent locations along two profiles. These models reveal a stratified subsurface structure consisting of six layers with varying resistivity and thickness. The uppermost layer exhibits resistivity values ranging from 241.1 to 4257 Ω m, followed by subsequent layers with resistivities of 78.81–2422 Ω m, 5.45–5267 Ω m, 49.72–263.3 Ω m, 7.96–303.3 Ω m, and 7.74–415 Ω m, respectively. Correspondingly, the thicknesses of these layers vary between 0.21–0.94 m, 0.11–0.76 m, 2.37–17.61 m, 6.08–12.79 m, 31.6–63.71 m, while their depths from the surface range from 0.21–0.94 m, 0.44–1.29 m, 3.57–18.43 m, 13.95–27.91 m, and 50.48–87.48 m, respectively.

#### Spatial constrained inversion (SCI-VES) results

Figures 11c and 12c illustrate the SCI-DC inversion models, utilizing a two-coupling width for model parameters between adjacent sites along the two primary profiles depicted in Fig. 1c. The SCI-DC inversion results reveal Six geo-electrical layers with distinct resistivity and thickness characteristics. The first layer exhibits variable resistivity (127.7–3781 Ω m) and thickness (0.17–0.97 m). The second layer shows resistivity values of 40.26–2688 Ω m, thickness ranging from 0.14 to 1.59 m, and depth between 0.17 and 0.97 m, while the third layer has resistivity values of 5.09–3401 Ω m, thickness of 2.23–14.85 m, and depth of 0.31 m. The first three geoelectrical layers comprise a dry, heterogeneous surface layer dominated by sand, sandy gravel, and basement fragments. The significant decrease in resistivity from 3781 Ω m at VES-05 to 127.7 Ω m at VES-02 near the coastline can be attributed to the increased presence of fine particles, particularly clay intrusions, within the geological strata. These clay-rich materials enhance conductivity, thereby reducing resistivity. Layers II and III display medium-to-low resistivity (5.09–47.33 Ω m) at specific locations (VES-02 and VES-03), suggesting clay intrusions or fractured basement aquifers. These layers vary in thickness from approximately 2.5 to 17.5 m, with the maximum thickness observed at VES-02.

The second geological layer, corresponding to the fourth geoelectrical layer, exhibits resistivity values ranging from 253.8 Ω m (VES-05) to 295.5 Ω m (VES-06) along the first profile, with a decreasing trend toward the southeast, as evidenced by lower values of 12.92 Ω m and 41.45 Ω m at VES-02 and VES-03, respectively. Along the second profile, resistivity varies between 298.1 Ω m (VES-04) and 384.1 Ω m (VES-09), peaking at 549.5 Ω m at VES-07. This layer’s thickness spans 11.16 m to 18.29 m, occurring at depths from 3.75 m (VES-08) to 15.92 m (VES-02). Composed of Quaternary-age freshwater-saturated gravelly sand deposits, the layer’s resistivity and thickness variations reflect its hydrogeological and stratigraphic characteristics.

The third geological layer, corresponding to the fifth geoelectrical layer, comprises brackishness-saturated clayey-sand deposits. Along the first profile, its resistivity varies between 64.09 Ω m (VES-06) and 260.4 Ω m (VES-04), while along the second profile, it ranges from 104.3 Ω m (VES-07) to 384.5 Ω m (VES-08). Notably, a decreasing resistivity trend is observed below VES-02 (4.53 Ω m) and VES-03 (13.83 Ω m) along the first profile. The layer’s thickness spans 17.34–72.68 m, with depths ranging from 15.72 m (VES-08) to 28.51 m (VES-02).

The geoelectric layer VI, representing a fractured basement aquifer saturated with saline water, exhibits resistivity values ranging from 15.09 to 77.26 Ω m along the first profile (VES-02 to VES-03) and from 33.88 to 137.1 Ω m along the second profile (VES-08 to VES-04), with an anomalously high value of 496.1 Ω m at VES-09. Depths for this layer vary between 33.06 m (VES-08) and 90.38 m (VES-06). These characteristics suggest significant seawater intrusion into the study area.

Notably, the resistivity trends in layers III, IV, and V mirror this pattern, decreasing toward the shoreline near VES-02 and VES-03, which underscores the influence of the uplifted, fractured basement aquifer (layer VI) on regional hydrogeological conditions.

Table 7 presents the output parameters of DC resistivity data, including resistivity, thickness, and depth, derived from 1D-DC, LCI-DC, and SCI-DC inversions along these profiles, highlighting their interdependent relationships.

**Table 7:** The table shows the number of water wells within the study area, the total dissolved solids (TDS) concentration, and the water depth from groundwater sample data.

Water Wells	Aquifer type	Latitude	Longitude	TDS (mg/l)	Water depth (m)	GWPZ
W1	QA	22.68	36.08	8386	1.35	Low
W2	QA	22.68	36.07	9750	1.05	Very low
W3	FBA	22.31	35.94	3522	28.35	Very high
W4	FBA	22.16	36.64	19,375	20.3	Moderate
W5	FBA	22.05	36.54	817	18.09	Moderate
W6	FBA	22.05	36.53	1139	10.6	Moderate
W7	FBA	22.03	36.48	1027	27	Moderate
W8	FBA	22.02	36.37	4289	29.5	Low
W9	FBA	22.12	36.35	2079	26	Very low
W10	FBA	22.15	36.43	4986	22	Moderate
W11	FBA	22.27	36.51	6151	8	Moderate

(QA) denotes the Quaternary aquifer while (FBA) denotes the Fractured basement aquifer.

#### Resolution of the constrained model parameters

The LCI-DC inversions yielded model parameters (resistivity and thickness) with varying resolutions along two profiles. Along the first profile, these parameters were resolved well to moderately (STDF = 1.010–1.491), while along the second profile, resolution ranged from well to poorly (STDF = 1.025–1.812). Notably, the resistivity of the second layer (Res.2) on the first profile and the thickness of the fourth layer (Th.4) across both profiles exhibited moderate resolution (STDF = 1.207–1.431). In contrast, the resistivity and thickness of the second layer (Res.2 & Th.2) along the second profile were poorly resolved due to higher STDF values (> 1.5; 1.534–1.812).

Conversely, SCI-DC inversions demonstrated superior resolution (nearly perfect or well resolved) for all parameters, with STDF values of 1.007–1.185 and 1.025–1.172 along the respective profiles. The consistently lower STDF values in SCI-DC inversions indicate enhanced information transfer between soundings along and across the profile, enabling superior parameter resolution compared to the LCI-DC method.

The sensitivity analysis of model parameters from the SCI-DC inversion demonstrates high resolution (STDF < 1.2) along two profiles, outperforming the LCI-DC inversion. This indicates that SCI-DC effectively reduces parameter uncertainty by fitting the data more accurately, aided by its smoother model space. The superior resolution and reduced uncertainty in SCI-DC inversions are attributed to their enhanced ability to constrain model parameters compared to LCI-DC methods*.* Consequently, this study adopts SCI results utilizing two coupling widths, as they provide the most reliable outcomes based on comparative analyses.

#### Comparison between 1D-VES, LCI-VES and SCI-VES results

Single-site inversion struggles to define lateral boundaries effectively due to data instability and noise, resulting in reduced lateral continuity. In contrast, LCI-DC and SCI-DC inversions produce resistivity and thickness distributions that differ from 1D inversion outcomes because of applied constraints on model parameters. These methods enhance subsurface resolution, making boundaries more detectable and reducing uncertainty in model parameters, thereby offering superior parameter resolution compared to 1D-DC inversion. The interplay between constraint application and increased resolution underscores their complementary roles in improving geoelectrical modeling accuracy.

LCI-DC and SCI-DC techniques enhance lateral continuity and subsurface resolution by incorporating lateral, vertical, and spatial constraints, enabling information exchange between adjacent measurements in multiple directions. This enables the resolution of ambiguities inherent in single-site inversions, where model parameters are often poorly defined. Specifically, LCI improves continuity along profiles by applying horizontal constraints, whereas SCI extends this capability to detect boundaries more comprehensively through spatially defined constraints, allowing parameter information to propagate across the entire profile rather than being restricted to linear pathways. Thus, the interplay of these techniques optimizes resolution and continuity, leveraging their complementary strengths in constraining subsurface models.

SCI-DC models offer more continuous and probable subsurface boundary representations, aligning well across all profiles and revealing geological units with greater detail compared to LCI-DC models. However, the lateral continuity of the deepest boundary diminishes in both SCI-DC and LCI-DC models between VES-07 and VES-09 along the second profile, likely due to normal fault influences. This highlights the superiority of 2D/3D inversions over 1D-DC inversion, as they better capture geological variations through stronger constraints between adjacent parameters.

Overall, the SCI-DC inversion provides the most reliable and realistic results, effectively restoring the area’s actual geology and producing coherent images, outperforming both 1D-DC and LCI-DC inversions.

### 2D-VES inversion results

To elucidate the spatial distribution of rock units and delineate geological structures, two 2D-inversion profiles of DC electrical resistivity data were constructed. These profiles, illustrated in Figs. 11d and 12d, reveal four distinct layers characterized by varying resistivity values. Such variations are predominantly influenced by differences in lithology, which encompasses gravel, sandy gravel, sand, clayey sand with clay intercalations from Pleistocene sediments, and the Precambrian fractured basement complex.

The inverted sections reveal a stratigraphic sequence characterized by distinct resistivity patterns. The uppermost layer comprises sandy-gravel deposits with high resistivity values (250 Ω m), while the second layer, consisting of sand or gravelly-sand saturated with freshwater, exhibits resistivity ranging from 100 to 140 Ω m. Notably, two sections display a very high resistivity anomaly (> 140 Ω m), indicative of gravel intrusions within this layer. The third layer, representing clay-sand deposits, shows resistivity varying between 25 and 60 Ω m along the first section and 90–125 Ω m along the second section. The lowermost layer, with decreasing resistivity (10 Ω m along the first section and 70 Ω m along the second section), suggests the presence of a fractured basement aquifer influenced by seawater intrusion. This conductive layer corresponds to the saline zone of the aquifer impacted by seawater intrusion. Notably, the third and fourth layers exhibit higher conductivity in the first profile compared to the second, attributable to their greater proximity to the coast, which exacerbates seawater intrusion in this area.

Lateral variations in resistivity values are attributed to basement uplift in the deeper sections, revealing structural complexity. Notably, basement positions beneath VES locations 2, 3, and 7, which lie along two perpendicular profiles, indicate intricate geological configurations. These variations are likely influenced by oblique dip-slip faults trending NE-SW along the first section and NW–SE along the second, as well as graben structures between VES-05 and VES-06 along the first section.

### Influence of geological structures on groundwater potentiality

The Red Sea Rift’s complex fault systems, play a critical role in shaping groundwater dynamics. These fault networks enhance porosity and secondary permeability, creating pathways for groundwater flow and storage within fault zones. In the study area, faulted aquifers influence surface boundaries and delineate potential groundwater zones, with high lineament density areas, particularly in the southwestern region (Fig. 6b), indicating significant groundwater potential. The presence of northeast-southwest and northwest-southeast trending strike-slip faults reveals complex structural controls that significantly influence groundwater flow, directing it upward toward specific regions and thereby impacting aquifer distribution and water quality. These tectonic features are corroborated by variations in resistivity values from DC resistivity data, which indicate transitions between saline and freshwater zones as well as changes in rock properties. The inverted 2D VES profiles further highlight these structural complexities, particularly in the central study area, where high-potential groundwater storage is evident. The interplay between these tectonic features and hydrogeological processes underscores their interconnected impact on regional groundwater resources and ensuring their sustainable management.

### Validation techniques used for groundwater potential model

The GIS-based AHP-derived groundwater potential zone (GWPZ) model was validated using hydrogeological data from eleven wells within the study area, incorporating well locations, total dissolved solids (TDS) concentrations, and groundwater depths (Fig. 9). The model’s accuracy was further assessed through a receiver operating characteristic-area under curve (ROC-AUC) analysis based on existing well distributions.

Notably, four wells located in low to very low potential zones. Six wells were situated in medium-potential areas, while a single well was identified in a very high-potential zone. The verification process involved comparing total dissolved solids (TDS) levels in the well samples with those of potential surrounding areas. In the Quaternary basin, TDS concentrations range from 8386 mg/L (W1) to 9750 mg/L (W2), significantly higher than the fractured aquifer’s range of 817 mg/L (W5) to 6151 mg/L (W11). Notably, Well 4 (W4) exhibits an exceptionally high TDS value of 19,375 mg/L. This disparity indicates greater salinity in groundwater samples (W1 and W2) from the Quaternary aquifer, attributed to seawater intrusion, as these wells are situated closer to the coast compared to those in the fractured aquifer (W3-W11).

Despite being located in a moderate zone according to the GWPZ map, Well 4 demonstrates elevated TDS levels due to its proximity to the coastline, which facilitates increased saltwater intrusion. From the comparison between Figs. 1 and 9, Wadi Diit extends from the southwest to the northeast towards the Red Sea coast, which penetrates the area with moderate to very high potential (yellow to dark green zones). Although Well 3 (W3) is located in an area with high potential, the values of dissolved solids in it are higher (3522 mg/L) because this well is located inside the valley where the floods sweep away all the sediments and dissolve the salts. As a result of their high infiltration into the ground, they lead to an increase in dissolved solids in the groundwater. Although W10 and W11 are located in a moderate zone, their samples have high salinity values due to their proximity to the valley floor. Also, the amount of total dissolved solids may also increase due to temperature and the mechanical energy of water movement through the formation.

There is a notable concordance between well sites 8 and 9 in the low to very low potential zones, characterized by high total dissolved solids (TDS) concentrations of 4289 mg/L and 2079 mg/L, respectively. Similarly, wells 5, 6, and 7 exhibit a strong correlation within medium-potential areas, featuring groundwater salinity levels ranging from low to moderate (817–1139 mg/L).

The groundwater aquifers in the study area exhibit varied salinity patterns, primarily shaped by geological and geographical factors. Notably, the Quaternary aquifer demonstrates elevated salinity levels (W1 & W2) due to substantial saltwater intrusion in coastal regions, which predominantly affects areas characterized by low to very low recharge potential. In contrast, most wells in the fractured basement aquifer are situated in regions of medium to very high potential, except for W8 and W9, which are located in low to very low potential zones. The total dissolved solids (TDS) analysis further indicates that the majority of these wells exhibit saline water quality (1027–6151 mg/L), with only W5 providing fresh water (817 mg/L) and W4 displaying exceptionally high salinity (19,375 mg/L). The fractured aquifer shows variable salinity linked to surrounding rock types; higher levels occur near metamorphic and volcanic metamorphic deposits, contrasting with lower salinity adjacent to granitic and ophiolite formations*.*

According to the GWPZ map, very high and moderate potential zones are predominantly inland or in southwestern regions, reducing their vulnerability to seawater intrusion relative to coastal areas. These interdependent factors highlight the complex relationship between geology, geography, and salinity distribution in the aquifer system.

The water depth in the Quaternary reservoir within wells 1 and 2 varies between 1.05 and 1.35 m, while that of the fractured basement reservoir ranges from 8 m (well 11) to 29.5 m (well 8). As indicated in Table 7, the water depth in the fractured basement reservoir is relatively shallow in areas of medium potential, such as wells 6 (10.5 m) and 11 (8 m), and progressively increases westward, away from the coast. This trend is attributed to variations in the thickness of the sedimentary cover, which is influenced by the complex structural elevation of the reservoir. These interdependent factors highlight the intricate relationship between geological structure and hydraulic characteristics in the studied area.

**Fig. 13:**
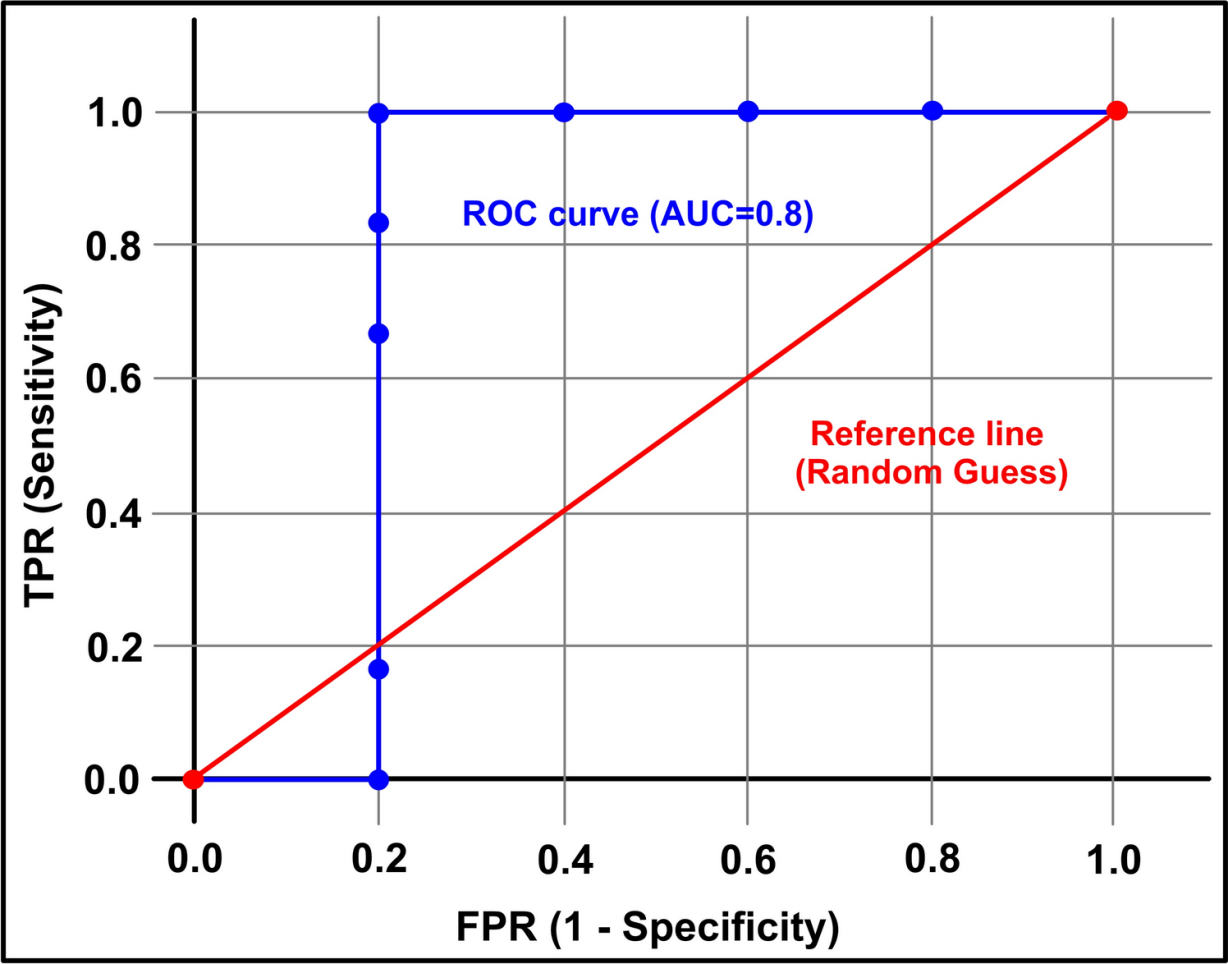
ROC-AUC curve for GWPZ map. (The figure was enhanced using Surfer 13.6 (www.goldensoftware.com), respectively, by Mohamed Genedi).

Additionaly, the groundwater recharge maps were validated using the AUC-ROC curve, as depicted in Fig. 13. Typically, models with AUC values between 0.7 and 0.9 are considered good, while those exceeding 0.9 are deemed excellent. The ensemble model achieved an AUC value of 0.8, indicating a good correlation between the groundwater potential zones (GWPZs) map and the locations of selected wells. This result confirms the validity of the GWPZs, suggesting a high likelihood of successful groundwater identification. Overall, these findings enhance confidence in the methodology as a robust framework for rapidly assessing groundwater recharge, thereby informing the placement of artificial recharge structures and supporting broader groundwater management strategies.

### Recommendations for decision makers on sustainable groundwater management

The study’s findings carry substantial policy implications for sustainable groundwater management in Egypt, emphasizing strategic prioritization of new well drilling sites in regions exhibiting medium to very high groundwater potential, particularly in the north-central area. These insights provide a scientific foundation for optimizing water resource allocation and guiding decision-makers in exploring and developing groundwater reserves within arid environments.

Simultaneously, the research underscores critical challenges such as saltwater intrusion and fluctuating recharge rates, revealing that areas with favorable recharge conditions, like those near coastal midlands, may still be vulnerable to contamination due to geological or anthropogenic factors. Thus, targeted investigations are essential to address these risks and ensure long-term sustainability in water resource management.

We recommend employing additional multi-criteria decision-making techniques, such as Multiple Influencing Factor (MIF) analysis, Analytic Network Process (ANP), and Fuzzy Analytical Hierarchy Process, to improve the identification of groundwater areas.

Constrained inversion techniques like LCI-DC and SCI-DC enhance surface resolution, enabling clearer insights into aquifer structures, thicknesses, depths, and the delineation of shallow Quaternary and fractured aquifers. These integrated approaches can guide decision-makers in optimizing groundwater exploration and development, particularly in arid regions like Wadi Diit, where water resources are vital for agriculture, industry, and drinking water supply.

## Conclusions

This study aims to delineate groundwater potential zones in the semi-arid Wadi Diit region of the southeastern desert by integrating conventional, remote sensing, and geophysical datasets. The area predominantly consists of Quaternary and Precambrian rocks, with lithosol and Yermosol-Hapalik soil types dominating the landscape. Annual rainfall data from 2021–2022 indicates a range of 15.64 mm/year to 30.30 mm/year, exhibiting higher values in the south that diminish progressively toward the north.

Sentinel-2 land use and land cover (LULC) data reveal that barren lands predominate in the region, with open shrublands and grasslands occupying limited areas. Topographic and hydrological characteristics, assessed through ASTER-GDEM data, indicate high to very high lineament densities confined to the southern and southwestern sections, signifying fractured basement structures in these zones. Furthermore, ASTER-L1B analysis demonstrates a spatial distribution of MI zones in the south and southwestern regions, contrasting with QI zones elsewhere.

A GIS-based Analytical Hierarchy Process (AHP) was employed to develop a groundwater potential (GWP) map for the study area, serving as a critical tool to achieve the research objectives. Nine thematic layers were integrated, derived from conventional data and ASTER-GDEM and ASTER-L1B remote sensing datasets, including qualitative index (QI), carbonate index (CI), slope, rainfall, drainage density, topographic wetness index (TWI), lineament density, land use/land cover (LULC), and mafic index (MI)*.* These layers were normalized, weighted, and ranked based on their influence on GWP, with QI identified as the most significant parameter. The model’s cross-validation accuracy was 0.0959, demonstrating its reliability in delineating potential zones.

The region was categorized into five potential zones: very low (4.53%), low (17.33%), moderate (27.05%), high (27.79%), and very high (23.3%). Notably, the north-central area exhibits significant potential due to its favorable geological and hydrological characteristics, including abundant quartz index deposits, Yermosols-type barren lands, medium to high rainfall, gentle topography, moderate to low drainage density and a well-developed drainage network with 5th and 6th stream orders and medium to high drainage density. These factors collectively enhance groundwater recharge capacity, making this region a priority for groundwater well drilling.

This study employed various unconstrained and constrained inversion techniques on DC resistivity data to enhance the lateral continuity of subsurface geological boundaries and delineate shallow Quaternary and fractured aquifers. While unconstrained 1D-DC inversion exhibited limited lateral continuity due to data ambiguity, the spatially constrained inversion (SCI-DC) and laterally constrained inversion (LCI-DC) offered improved resolution. The SCI-DC technique, by emphasizing spatial coherence, provided greater detail in continuous subsurface boundaries compared to LCI-DC, which focuses on lateral constraints. Furthermore, SCI-DC effectively reduced model parameter uncertainty, as its standard deviation factor (STDF) was well-resolved, thereby clarifying parameters poorly defined in 1D-DC and LCI-DC inversions. Consequently, SCI-DC results were selected for this research, as they yielded more reliable, realistic outcomes and better reconstructed the overall geological units.

SCI-DC inversion models identified six geo-electrical layers, interpretable as four distinct geological units across two profiles. These layers, from top to bottom, correspond to dry heterogeneous wadi deposits (127.7–3781 Ω m), gravelly-sand deposits saturated with fresh water (12.92–549.5 Ω m), clayey-sand deposits with brackish water saturation (4.53–384.5 Ω m), and fractured basement rocks (15.09–496.1 Ω m). The second layer transitioning from low to high resistive properties, represents the Quaternary aquifer, characterized by fresh water saturation, with a thickness of 11.16–18.29 m and depths ranging from 3.75 to 15.92 m. The lowest layer, exhibiting low to medium resistivity, signifies the fractured basement aquifer affected by seawater intrusion at depths of 33.06–90.38 m.

The 2D-VES inversion results reveal four distinct subsurface layers. The top layer consists of sandy-gravel deposits with resistivity ranging from 250 to 600 Ω m, followed by a second layer of either sand (100–140 Ω m) or gravelly-sand (> 140 Ω m), both saturated with fresh water. Below this lies a third layer of clayey-sand deposits, characterized by resistivity values of 25–125 Ω m, and a fourth layer comprising a saline-fractured basement aquifer with conductivities between 10 and 70 Ω m. Notably, the third and fourth layers exhibit higher conductivity along the first profile compared to the second, indicating seawater intrusion influenced by coastal proximity.

The SCI-VES and 2D-VES models reveal a lateral decrease in resistivity values toward the coastline, potentially indicating shallow clay intrusions or deeper influences from an uplifted, fractured basement aquifer. This observation suggests a complex 2D/3D geological structure, likely shaped by dip-slip faults trending NE-SW and NW–SE. These fault systems serve as conduits for groundwater flow toward the upper sections of the study area.

From the comparison between GWPZ and locations of wells, most of them were situated in medium to very high potential areas.The ROC-AUC curve demonstrated a good correlation (AUC = 0.8) between the GWPZ map and the selected well locations, validating the accuracy of the suitability model for identifying potential groundwater areas*.* The consistency ratio (CR = 0.0956) further confirmed the reliability of the criteria weights and suitability levels employed in the model. These robust validation outcomes underscore the effectiveness of the methodology as a rapid assessment framework for groundwater recharge, providing decision-makers with essential information while enhancing confidence in its application.

## Data Availability

The conventional and remote sensing datasets utilized in this study can be obtained from the corresponding author upon reasonable request. For reference, an example of ASTER-GDEM data is included in the manuscript, accessible via the following link: (https://search.earthdata.nasa.gov/search). These datasets collectively underpin the analysis, highlighting their interdependent roles in enhancing the study’s outcomes.
